# Weak endogenous Ca^2+^ buffering supports sustained synaptic transmission by distinct mechanisms in rod and cone photoreceptors in salamander retina

**DOI:** 10.14814/phy2.12567

**Published:** 2015-09-28

**Authors:** Matthew J Van Hook, Wallace B Thoreson

**Affiliations:** 1 Truhlsen Eye Institute and Department of Ophthalmology & Visual Sciences, University of Nebraska Medical CenterOmaha, Nebraska; 2 Department of Pharmacology & Experimental Neuroscience, University of Nebraska Medical CenterOmaha, Nebraska

**Keywords:** Calcium, photoreceptor, retina, ribbon synapse, synaptic transmission

## Abstract

Differences in synaptic transmission between rod and cone photoreceptors contribute to different response kinetics in rod- versus cone-dominated visual pathways. We examined Ca^2+^ dynamics in synaptic terminals of tiger salamander photoreceptors under conditions that mimicked endogenous buffering to determine the influence on kinetically and mechanistically distinct components of synaptic transmission. Measurements of *I*_C__l(Ca)_ confirmed that endogenous Ca^2+^ buffering is equivalent to ˜0.05 mmol/L EGTA in rod and cone terminals. Confocal imaging showed that with such buffering, depolarization stimulated large, spatially unconstrained [Ca^2+^] increases that spread throughout photoreceptor terminals. We calculated immediately releasable pool (IRP) size and release efficiency in rods by deconvolving excitatory postsynaptic currents and presynaptic Ca^2+^ currents. Peak efficiency of ˜0.2 vesicles/channel was similar to that of cones (˜0.3 vesicles/channel). Efficiency in both cell types was not significantly affected by using weak endogenous Ca^2+^ buffering. However, weak Ca^2+^ buffering speeded Ca^2+^/calmodulin (CaM)-dependent replenishment of vesicles to ribbons in both rods and cones, thereby enhancing sustained release. In rods, weak Ca^2+^ buffering also amplified sustained release by enhancing CICR and CICR-stimulated release of vesicles at nonribbon sites. By contrast, elevating [Ca^2+^] at nonribbon sites in cones with weak Ca^2+^ buffering and by inhibiting Ca^2+^ extrusion did not trigger additional release, consistent with the notion that exocytosis from cones occurs exclusively at ribbons. The presence of weak endogenous Ca^2+^ buffering in rods and cones facilitates slow, sustained exocytosis by enhancing Ca^2+^/CaM-dependent replenishment of ribbons in both rods and cones and by stimulating nonribbon release triggered by CICR in rods.

## Introduction

**Figure 1:**
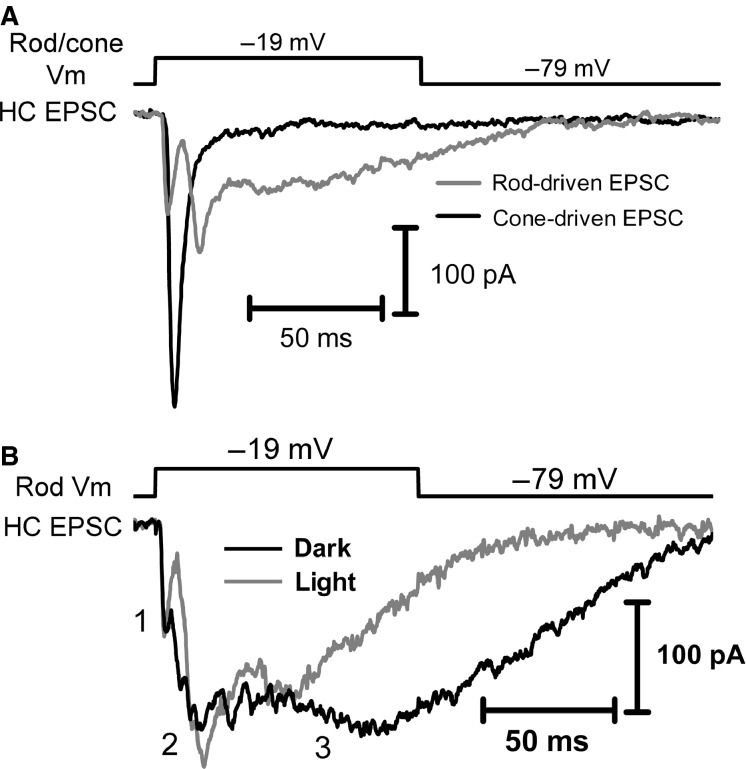
Differences in rod- and cone-driven postsynaptic currents. (A) EPSCs recorded in a single horizontal cell driven by either a presynaptic cone (black trace) or a rod (gray trace) when the photoreceptors were stimulated with 100 msec duration voltage steps (to –19 mV from –79 mV). After recording cone-driven responses, the patch pipette was removed from the cone and a new pipette was used to establish a recording from a nearby rod. The differences in EPSC kinetics are largely the result of differences in presynaptic mechanisms rather than postsynaptic properties, as both rod- and cone-driven synapses in salamander rely on AMPA receptors (Cadetti et al. 2005), although differences in AMPA receptor subunit composition might make subtle contributions to the differences. (B) A rod-driven EPSC from a different rod-HC paired recording. The rod-driven EPSC exhibits the following three major components: (1) efficient phasic release of vesicles from the synaptic ribbon; (2) CICR-driven nonribbon release; and (3) release from neighboring rods excited via gap junctional coupling to the recorded rod. Component #3 is reduced when neighboring rods are hyperpolarized by an acutely delivered flash of light (gray).

In the vertebrate retina, rod and cone photoreceptors differ dramatically in their kinetics of synaptic transmission (Fig. 1), with rod transmission being considerably slower than transmission by cones (Pasino and Marchiafava 1976; Baylor and Fettiplace 1977; Schnapf and Copenhagen 1982; Copenhagen et al. 1983; Witkovsky and Stone 1983; Witkovsky et al. 1997; Cadetti et al. 2005; Rabl et al. 2005; Thoreson 2007). These differences in synaptic transmission parallel rod/cone differences in light response kinetics and influence response kinetics in rod- and cone-driven retinal circuits (Korenbrot and Rebrik 2002). Although differing in kinetics, both photoreceptor types share the property that transmission involves the synaptic ribbon, a protein structure that tethers glutamate-filled synaptic vesicles near the presynaptic membrane and readies them for exocytosis (Heidelberger et al. 2005). The overall slower transmission kinetics of rods arises from substantial additional contributions of release from nonribbon sites triggered by the release of Ca^2+^ from intracellular stores via CICR (Cadetti et al. 2006; Babai et al. 2010b; Chen et al. 2013, 2014).

Ca^2+^ regulates synaptic vesicle exocytosis from rods and cones by multiple mechanisms and so the handling of Ca^2+^ ions by buffering, sequestration, and extrusion in each photoreceptor type plays an important role in regulating the fine structure of light response encoding in rod- and cone-driven visual pathways. We have previously used the “added buffer approach” (Neher and Augustine 1992; Helmchen et al. 1997; Van Hook and Thoreson 2014) to measure the endogenous Ca^2+^ binding ratio of buffers at rod and cone synaptic terminals, finding that buffering was relatively low and similar in both photoreceptor types (*κ*’ = 30 in rods and 50 in cones). This Ca^2+^ binding ratio is equivalent to approximately 0.05–0.1 mmol/L EGTA. For comparison, buffering at the synapses of ribbon-bearing hair cells and rod bipolar cells has been measured to be equivalent to approximately 1–2 mmol/L EGTA or BAPTA (Burrone et al. 2002; Singer and Diamond 2003; Frank et al. 2009; Mehta et al. 2014). In the present study, we analyzed the effects of low endogenous Ca^2+^ buffering on the different components of release from rods and cones and evaluate the extent to which buffering shapes rod/cone differences in response kinetics.

Studies in cones show that Ca^2+^ influx through L-type Ca^2+^ channels at the ribbon base triggers fast phasic vesicle release from an immediately releasable pool (IRP), followed by slower release of a second reserve pool (Bartoletti et al. 2010) (Fig. 1A). The cone IRP corresponds in size to the population of vesicles along the bottom two rows of the ribbon (˜15–20 vesicles) and the reserve pool corresponds to the remaining vesicles populating the ribbon (Bartoletti et al. 2010). During sustained depolarization of cones, the rate of release is limited by the rate at which vesicles can be resupplied to ribbon release sites (Babai et al. 2010a; Van Hook et al. 2014). Ca^2+^ acting through calmodulin (CaM) accelerates this resupply process by acting on ribbon-associated proteins to enhance the likelihood of vesicle attachment (Van Hook et al. 2014). One question posed in this study is whether the mechanisms regulating sustained and transient release of vesicles from ribbons are similar in rods and cones. To answer this question, we measured the IRP, release efficiency, and influence of CaM on release in rods.

Blocking CICR can reduce rod-driven postsynaptic responses by >50% in mammalian and amphibian retina showing that this mechanism is a major contributor to sustained release from rods in darkness (Suryanarayanan and Slaughter 2006; Babai et al. 2010b). Previous evidence obtained with relatively strong Ca^2+^ buffering suggested that CICR does not occur in cone terminals and that all release from cones instead occurs at the synaptic ribbons (Krizaj et al. 2003; Snellman et al. 2011). Does CICR emerge in cones with low endogenous buffering? In ribbon-bearing retinal bipolar cells that do not exhibit CICR, a slow component of nonribbon release can be revealed after saturating presynaptic buffers with a large Ca^2+^ load (Mehta et al. 2014). Can slower nonribbon components of release also be triggered in cones given sufficient elevation of intraterminal [Ca^2+^]?

To address these questions, we combined imaging studies of intraterminal Ca^2+^ levels and electrophysiological measurements of synaptic release from rods and cones in the salamander retina. We determined that ribbon-mediated release in rods operates similarly to release from cones although rods showed slightly slower kinetics and slightly lower release efficiency. After confirming that buffering in rod and cone terminals was equivalent to approximately 0.05 mmol/L EGTA, we also found that the use of weak EGTA buffering had no effect on the peak efficiency of IRP release in either rods or cones. This lack of an EGTA effect is a hallmark of nanodomain control of exocytosis (Eggermann et al. 2012). While it did not alter fast release, low endogenous buffering enhanced slow release. In both rods and cones, low Ca^2+^ buffering accelerated Ca^2+^/CaM-dependent replenishment of vesicles to ribbons. Additionally, in rods but not cones, low endogenous buffering enhanced CICR and thereby increased slow release at nonribbon sites. Even with weak buffering, we found no evidence for CICR in cone terminals. Furthermore, enhancing the intraterminal spread of Ca^2+^ by low buffering and inhibition of Ca^2+^ extrusion did not recruit additional slow components of release in cones. In addition to confirming that release from cones occurs exclusively at ribbons, this shows that rods possess specialized capabilities for nonribbon release that are not present in cones.

## Materials and Methods

### Ethical approval

All experiments were performed using vertical slices from retinas of aquatic tiger salamanders (*Ambystoma tigrinum*), 18–25 cm in length (Charles Sullivan, Nashville, TN). Animal housing and protocols were approved by the Institutional Animal Care and Use Committee at the University of Nebraska Medical Center. Euthanasia was conducted in accordance with AVMA Guidelines for the Euthanasia of Animals.

### Retinal slices and electrophysiology

Salamanders were housed at 8°C on a 12-h light/dark cycle. Animals were immersed in the anesthetic MS222 (0.25 g/L) for 15 min, 1–2 h after the beginning of the dark cycle. They were then decapitated, quickly pithed, and enucleated. Retinas were mounted on a nitrocellulose membrane (type AAWP, 0.8 *μ*m pores; Millipore) and cut into 125 *μ*m vertical slices using a razor blade tissue chopper (Stoelting), as described previously (Van Hook and Thoreson 2013). Slices were rotated 90 degrees to view the retinal layers and mounted in a recording chamber that was positioned on an upright fixed-stage microscope (Nikon E600FN) equipped with a 60× water immersion objective. Slices were superfused at ˜1 mL/min with an oxygenated amphibian saline solution containing (in mmol/L) 116 NaCl, 2.5 KCl, 1.8 CaCl_2_, 0.5 MgCl_2_, 5 glucose, and 10 HEPES. The pH was adjusted to 7.8 with NaOH and the osmolality was measured with a vapor pressure osmometer (Wescor) and adjusted to 245 mOsm. Experiments were performed ˜2–8 h after the beginning of the dark cycle. Previous studies have indicated that photoreceptor ribbon morphology changes throughout the day in both mouse and turtle, with ribbons maintaining their classical plate-like morphology during the hours over which we conducted experiments (Abe and Yamamoto 1984; Adly et al. 1999).

For paired whole cell recordings, we targeted photoreceptors and horizontal cells based on soma position and morphology. Cell identity was confirmed based on physiological criteria (Van Hook and Thoreson 2013) and, in some cases, by imaging of fluorescent dyes included in the pipette solution. Patch pipettes were pulled from borosilicate glass (1.2 mm OD, 0.9 mm ID, with an internal filament; World Precision Instruments). Pipette tip diameter was 1–2 *μ*m and resistance was typically 15–20 MΩ. The photoreceptor patch pipette was positioned on the ellipsoid in the inner segment of rods and cones and the horizontal cell pipette was placed on the soma. The horizontal cell (postsynaptic) patch pipette contained (in mmol/L) 90 Cs-gluconate, 10 TEA-Cl, 3.5 NaCl, 1 MgCl_2_, 1 CaCl_2_, 0.5 GTP-Na, 9.6 ATP-Mg, and 10 HEPES. The photoreceptor (presynaptic) pipette solution contained (in mmol/L) 50 Cs-gluconate, 40 Cs-glutamate, 10 TEA-Cl, 3.5 NaCl, 1 MgCl_2_, 10 ATP-Mg, 0.5 GTP-Na, 10 HEPES, and 0.05 EGTA. The use of 0.05 mmol/L EGTA was intended to approximate the endogenous Ca^2+^ buffering present in photoreceptor terminals (Van Hook and Thoreson 2014). When we instead used a presynaptic pipette solution containing 5 mmol/L EGTA for paired recordings, it also contained 1 mmol/L CaCl_2_. As indicated in the Results section, we sometimes included 1 mmol/L sodium orthovanadate (Na_3_VO_4_) in the photoreceptor pipette solution to inhibit the plasma membrane Ca^2+^ ATPase (PMCA) (Morgans et al. 1998). In some recordings, we included Sulforhodamine-B (1 mg/mL) and Lucifer yellow (2 mg/mL) in the pre- and postsynaptic pipette solutions, respectively. To count synaptic ribbons, we used a HiLyte488-conjugated peptide (80 μmol/L) containing a PXDLS sequence that binds to the B domain of Ribeye, the major ribbon protein, and introduced this peptide through the rod patch pipette solution (Zenisek et al. 2004; Bartoletti et al. 2010). The pH and osmolality of the pipette solutions were measured and adjusted to 7.2 and 235–240 mOsm, respectively. Reported voltages were corrected for the liquid junction potential, which was measured as 9 mV.

When measuring the voltage dependence of Ca^2+^-activated Cl^–^ currents [*I*_Cl(Ca)_], the photoreceptor pipette solution was as above, except that it contained 5, 0.5, or 0.05 mmol/L EGTA and 0 added Ca^2+^ in all experiments. For perforated patch recordings, we used a pipette solution containing 200 μmol/L amphotericin-B and 5 *μ*g/mL gramicidin with 0.1 mmol/L EGTA and no added Ca^2+^. Amphotericin-B was diluted 1000× from a stock made in DMSO and gramicidin 1000× from a stock made in 95% ethanol. Fresh antibiotic solutions were made every 3 h and stored in the dark. We also included Lucifer yellow (2 mg/mL) or Sulforhodamine-B (1 mg/mL) in the perforated patch pipette solution. To establish perforated patch recordings, we waited for the appearance of whole cell capacitance transients after establishing a giga-ohm seal. Cells were typically fully perforated for 5–10 min. At the end of the recording, we confirmed that the patch had not ruptured by taking images showing that fluorescent dye did not fill the cell. We then applied suction to rupture the patch, which caused an increase in the amplitude of the whole cell capacitance transients and diffusion of dye into the cell.

In paired rod-horizontal cell (HC) recordings, a clear fast component of the excitatory postsynaptic current (EPSC), representing exocytosis of ribbon-associated vesicles is followed by two distinguishable slow components. These are illustrated in the examples of rod- and cone-driven EPSCs shown in Figure 1. Following fast ribbon-mediated release, the first of the two slow components of rod-driven release represents exocytosis at nonribbon release sites that is triggered by CICR (Krizaj et al. 1999; Cadetti et al. 2006; Suryanarayanan and Slaughter 2006; Chen et al. 2013, 2014). A final component of rod-driven EPSCs in salamander retina arises from the spread of depolarizing current into neighboring rods through gap junctions. This component can be reduced by gap junction inhibitors (Cadetti et al. 2006) and by hyperpolarizing nonvoltage-clamped rods with acute illumination (Fig. 1B). To minimize the contribution of release from electrically coupled rods, we briefly illuminated the retina while stimulating the rod with depolarizing test steps during paired recordings.

### Release efficiency measurements

Synaptic vesicle release efficiency – the number of voltage-gated calcium channel openings and the number of miniature EPSCs (mEPSCs) contributing to evoked EPSCs – was measured using a deconvolution approach, as described previously (Bartoletti et al. 2011). Deconvolution of the leak-subtracted calcium current (*I*_Ca_, measured with a P/8 protocol) and EPSC was performed using OriginPro8. *I*_Ca_ was deconvolved using a single channel probability density function with a peak amplitude of 0.31 pA and a decay time constant of 1.1 msec describing the properties of L-type Ca^2+^ channels in photoreceptor terminals (Thoreson et al. 2000; Bartoletti et al. 2011). EPSCs were deconvolved with an empirically measured mEPSC waveform with an amplitude of 5.7 pA and a charge of 15.5 fC (Cadetti et al. 2008). We calculated the number of ribbon contacts in each recorded pair by dividing the EPSC by 46 pA (Bartoletti et al. 2010) for cone-HC pairs and by 36 pA for rod-HC pairs (this study). The number of channel openings was scaled by an average number of 13 ribbons per cone (Pang et al. 2008; Bartoletti et al. 2010) and seven ribbons per rod (this study) (Townes-Anderson et al. 1985; Pang et al. 2008). Release events were shifted forward by 300 *μ*sec to compensate for latency in release following an instantaneous rise in Ca^2+^ concentration (Duncan et al. 2010). The deconvolution results were low-pass filtered using a fast Fourier transform filter with 5–10 points of smoothing. The rate of Ca^2+^ channel openings was divided by rate of release events to calculate the number of fusion events per channel opening at each time point.

### Imaging of Ca^2+^ signals in rod and cone terminals

Ca^2+^ imaging was performed during whole cell recordings in retinal slices by filling photoreceptors with the Ca^2+^ sensitive dye Oregon Green 488 BAPTA-5N (OGB-5N, *k*_d_ = 20 μmol/L; Invitrogen) included in the patch pipette solution (500 μmol/L). OGB-5N at this concentration contributes a Ca^2+^ binding ratio (*κ*’) of ˜20. When included with a pipette solution containing 0.05 mmol/L EGTA, this produces a total Ca^2+^ *κ*’ of ˜50, which is within the range of measurements we previously reported for both rods and cones (Van Hook and Thoreson 2014). Images were acquired on a Nikon E600FN microscope equipped with a spinning disk laser confocal scan head (PerkinElmer Ultraview LCI) and a cooled CCD camera (Orca ER) with UltraView Imaging Suite software (PerkinElmer). OGB-5N was excited with a 488-nm laser and emitted light passed through a 525-nm long-pass filter. The frame was cropped and binned 2 × 2 to the synaptic terminals to give quick acquisition speeds of 35 msec exposures at 35 msec intervals. Ca^2+^-dependent fluorescent signals were analyzed as Δ*F*/*F*_o_ (measured from a 1–2 sec prestimulus baseline) in response to depolarizing steps in voltage clamp (–79 to –19 mV) of varying duration (20–500 msec).

### Statistical analysis

Electrophysiology data were analyzed using Microsoft Excel, Clampfit 10.4, and GraphPad Prism 4.0 software. Ca^2+^ imaging data were analyzed with PerkinElmer Ultraview Imaging Suite software. Data are presented as mean ± standard error of the mean (SEM) of individual measurements from single cells or single pre- and postsynaptic recordings. Unless otherwise noted, differences were considered statistically significant with *P *<* *0.05 measured with a two-tailed independent Student’s *t*-test.

## Results

### Endogenous Ca^2+^ buffering in rods and cones

**Figure 2:**
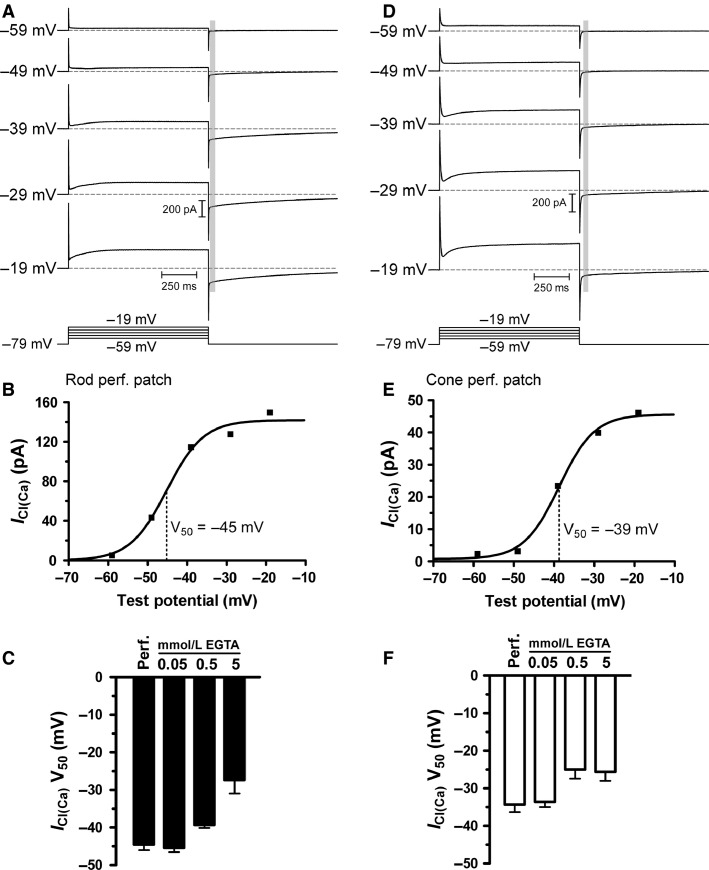
Effects of endogenous and exogenous Ca^2+^ buffers on Ca^2+^-activated Cl^–^ current activation. (A) *I*_C__l(Ca)_ activation in a perforated patch recording from a rod. *I*_C__l(Ca)_ amplitude was measured following depolarizing voltage steps (1 sec duration) to –59, –49, –39, –29, and –19 mV from a holding potential of –79 mV. *I*_C__l(Ca)_ was measured from the prestep baseline (dotted line) after the decay of the whole cell capacitance transient. The measurement region is indicated by the vertical gray bar. (B) Current–voltage relationship of *I*_C__l(Ca)_ from the rod in A, fit with a Boltzmann sigmoid. The voltage of half-maximal activation (*V*_50_) was –45 mV in this rod. (C) Group data comparing *I*_C__l(Ca)_ *V*_50_ measurements with different concentrations of the exogenous Ca^2+^ buffer EGTA or when endogenous buffers were kept intact by recording in perforated patch configuration. The perforated patch *V*_50_ most closely matches the 0.05 mmol/L EGTA condition. (D) *I*_C__l(Ca)_ recorded in a single cone using perforated patch techniques, as in A. (E) Current–voltage relationship showing a *V*_50_ for *I*_C__l(Ca)_ activation in this cone of –39 mV. (F) Group data showing that for cones, *V*_50_ recorded with perforated patch was most similar to the *V*_50_ recorded with 0.05 mmol/L EGTA.

The spatiotemporal dynamics of Ca^2+^ signals in the photoreceptor terminal are determined by a combination of Ca^2+^ influx, buffering, and extrusion and these dynamics shape the relative contributions of each of the Ca^2+^-dependent phases of exocytosis (Fig. 1). Using the added buffer approach (Neher and Augustine 1992; Helmchen et al. 1997), we previously found that the endogenous buffering in rod and cone photoreceptors exhibits a capacity equivalent to approximately 0.05 mmol/L EGTA (Van Hook and Thoreson 2014). This suggests that differences in kinetics of release at rod and cone synapses (Cadetti et al. 2005; Rabl et al. 2005) do not originate with differences in intraterminal Ca^2+^ buffering. We sought to verify measurements of the endogenous buffering capacity from changes in the voltage dependence of the calcium-activated chloride current (*I*_Cl(Ca)_) in rod and cone photoreceptors (Barnes and Deschênes 1992; MacLeish and Nurse 2007; Mercer et al. 2011) produced by varying the concentration of EGTA in the patch pipette solution in whole cell recordings or recording in perforated patch configuration to preserve endogenous buffers (Fig. 2). The properties of *I*_Cl(Ca)_ differ between rods and cones, which might be attributable to differences in *I*_Cl(Ca)_ properties between each photoreceptor type such as channel distance relative to Ca^2+^ channels and/or Ca^2+^ cooperativity and sensitivity (Mercer et al. 2011). For instance, previous work has shown that *I*_Cl(Ca)_ channels are located within several hundred nanometers of Ca^2+^ channels and the current has an EC_50_ for Ca^2+^ of approximately 550 nmol/L in rods and 380 nmol/L in cones (Mercer et al. 2011), although the cooperativity differs between the two photoreceptor types. Thus, increasing exogenous buffering with EGTA will shift the voltage dependence of *I*_Cl(Ca)_ activation by buffering progressively more Ca^2+^ near the *I*_Cl(Ca)_ channels. In this experiment, therefore, the meaningful comparison is between the voltage of half-maximal activation (*V*_50_) of *I*_Cl(Ca)_ at different levels of Ca^2+^ buffering within the population of rods or cones rather than a comparison of *I*_Cl(Ca)_ properties between rods and cones. A similar approach, which involved recording Ca^2+^-activated potassium currents, has been used to measure the endogenous buffering in retinal bipolar cells (Burrone et al. 2002). Rods and cones were voltage clamped at –79 mV and *I*_Cl(Ca)_ was evoked with a 1-sec long depolarization to test potentials ranging from –59 to –19 mV. The *V*_50_ shifted to more depolarized potentials as the concentration of EGTA in the pipette solution was increased in both rods (0.05 mmol/L EGTA, *V*_50_ = –45.4 ± 1.1 mV, *n* = 7; 0.5 mmol/L EGTA *V*_50_ = –39.3 ± 0.8, *n* = 5; 5 mmol/L EGTA, *V*_50_ = –27.4 ± 3.6 mV, *n* = 8) and cones (0.05 mmol/L EGTA, *V*_50_ = –33.6 ± 1.4, *n* = 9; 0.5 mmol/L EGTA, *V*_50_ = –25.0 ± 2.4, *n* = 7; 5 mmol/L EGTA, *V*_50_ = –25.6 ± 5.1 mV, *n* = 4). Using perforated patch recordings to preserve endogenous buffering, we found that the *V*_50_ was –44.5 ± 1.5 mV (*n* = 8) for rods and –34.3 ± 2.0 (*n* = 8) for cones. These values are quite similar to the *V*_50_ measurements obtained with the 0.05 mmol/L EGTA solution, indicating that this is a good approximation for the endogenous buffering in rod and cone synaptic terminals and supporting measurements made by the added buffer approach (Van Hook and Thoreson 2014).

### Spatiotemporal Ca^2+^ dynamics in rods and cones

**Figure 3:**
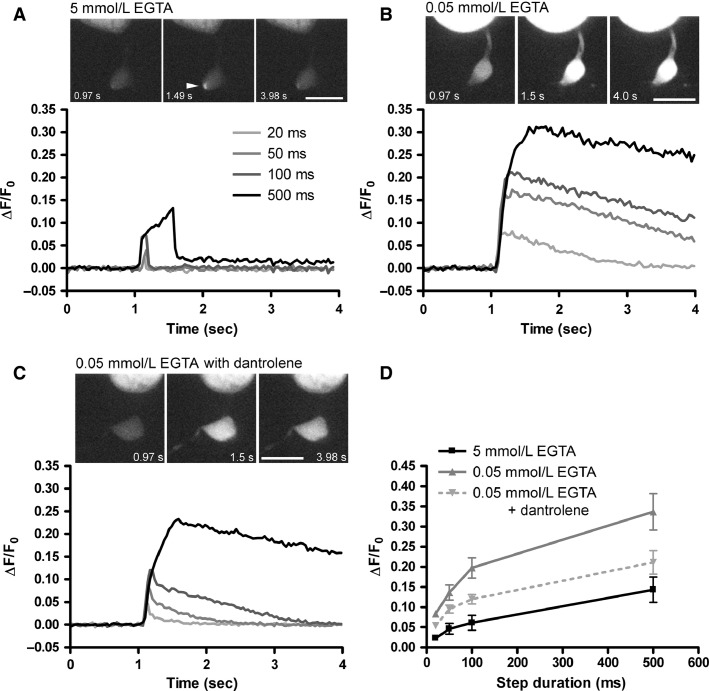
Endogenous levels of Ca^2+^ buffering promote CICR-mediated boosting of intraterminal Ca^2+^ signals in rods. (A) Fluorescent Ca^2+^ signals measured in the synaptic terminal of a rod dialyzed with 5 mmol/L EGTA and 500 μmol/L OGB-5N in response to depolarizing voltage steps (to –19 mV from a *V*_hold_ = –79 mV) of varying duration (20, 50, 100, and 500 msec). The peak Δ*F*/*F*_0_ amplitude increased with increasing step duration and the Ca^2+^ transients declined rapidly at the end of the voltage step. The top panel shows the OGB-5N fluorescence images in a single rod terminal before (0.97 sec), at the peak (1.49 sec), and after (3.98 sec) the 500 msec step. The fluorescence signal in this cell was constrained to a hotspot near the base of the terminal (arrowhead). Scale = 10 *μ*m. (B) The amplitudes of Ca^2+^ signals were enhanced and the recovery slowed with 0.05 mmol/L intracellular EGTA. Top panel showing the OGB-5N fluorescence images demonstrates that with 0.05 mmol/L EGTA, the OGB-5N fluorescence was stronger, was spread throughout the terminal, and was still elevated at the end of the acquisition period (4.0 sec). Scale = 10 *μ*m. (C) The enhancement with 0.05 mmol/L EGTA was inhibited by including 50 μmol/L dantrolene in the patch pipette of a different rod, consistent with a role for CICR in boosting intraterminal [Ca^2+^]. The top panel shows images of OGB-5N fluorescence within the rod terminal. Scale = 10 *μ*m. (D) Group data showing the increase in Δ*F*/*F*_0_ with increasing step duration, the enhancement with 0.05 mmol/L EGTA, and the inhibition with dantrolene.

**Figure 4:**
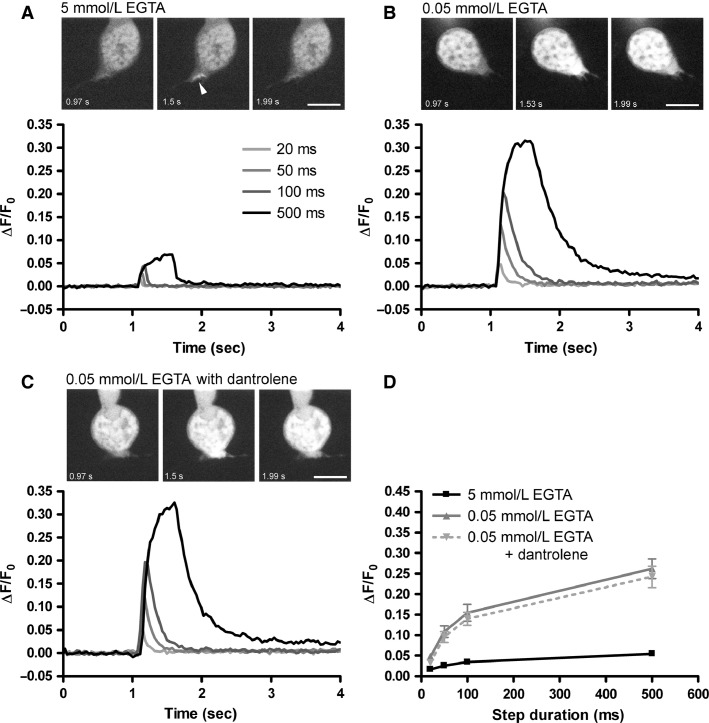
Endogenous levels of Ca^2+^ buffering enhance intraterminal Ca^2+^ signals in cones independently of CICR. (A) Ca^2+^ transients measured in the synaptic terminal of a cone dialyzed with 5 mmol/L EGTA in response to depolarizing voltage steps (to –19 mV from a *V*_hold_ = –79 mV) of varying duration (20, 50, 100, and 500 msec). The Ca^2+^ transients declined rapidly at the end of the voltage step and the peak Δ*F*/*F*_0_ amplitude increased with increasing step duration. The top panel shows OGB-5N fluorescence images acquired before (0.97 sec), at the peak (1.5 sec), and after (1.99 sec) the 500 msec stimulus. The fluorescence signal in this cell was constrained to a cluster of hotspots near the base of the terminal (arrowhead). Scale = 10 *μ*m. (B) In a different cone, the amplitudes of the Ca^2+^ signals were enhanced and recovery slowed with 0.05 mmol/L intracellular EGTA. The top panel shows the OGB-5N fluorescence images, demonstrating that with 0.05 mmol/L EGTA, the OGB-5N fluorescence was stronger and spreads throughout the terminal. Scale = 10 *μ*m. (C) As shown in a different cone, this enhancement with 0.05 mmol/L EGTA was not affected by including 50 μmol/L dantrolene in the patch pipette. The top panel shows the OGB-5N fluorescence images. Scale = 10 *μ*m. (D) Group data showing the increase in Δ*F*/*F*_0_ with increasing step duration, the enhancement with 0.05 mmol/L EGTA, and the lack of an effect of dantrolene on the enhanced Ca^2+^ signal.

We next performed Ca^2+^ imaging experiments to determine how conditions that mimic the endogenous buffering influence the spatiotemporal dynamics of Ca^2+^ signals in rod (Fig. 3) and cone (Fig. 4) synaptic terminals. To accomplish this, we loaded photoreceptors with the low affinity Ca^2+^ dye Oregon Green BAPTA 5N (OGB-5N, 500 μmol/L, *k*_d_ = 20 μmol/L) through the patch pipette using solutions containing either 0.05 mmol/L or 5 mmol/L EGTA. Ca^2+^ influx was evoked by step depolarizations (to –19 mV) of 20, 50, 100, or 500 msec, which mimics the response to a transition to darkness following a strong flash of light in photoreceptors. Under both buffering conditions, the amplitude of the Ca^2+^ signal increased with increasing step duration and, when mimicking endogenous buffering by using 0.05 mmol/L EGTA, the Ca^2+^ signals were greater in amplitude, as expected. With 100 msec steps in rods (Fig. 3), for instance, Δ*F*/*F*_0_ was increased from 0.06 ± 0.018 (*n* = 6) with 5 mmol/L EGTA to 0.197 ± 0.025 with 0.05 mmol/L EGTA (*n* = 9, *P *=* *0.0007). In cones (Fig. 4), responses to 100 msec steps were increased from Δ*F*/*F*_0_ of 0.034 ± 0.005 (*n* = 8) with 5 mmol/L EGTA to 0.155 ± 0.020 (*n* = 6) with 0.05 mmol/L EGTA (*P *=* *0.0016). Increased Ca^2+^ buffering also constrained the Ca^2+^ signal to a hotspot, reflecting influx through Ca^2+^ channels located beneath a single ribbon or a few ribbons. With 0.05 mmol/L EGTA, in contrast, the fluorescent Ca^2+^ signal spread throughout the terminal. We quantified this by measuring the ratio of Δ*F*/*F*_0_ in the terminal to Δ*F*/*F*_0_ at the hotspot (Ca_terminal_/Ca_hotspot_). In rods, Ca^2+^ was largely restricted to sites of entry in the terminal with 5 mmol/L EGTA (ratio = 0.11 ± 0.02, *n* = 20) and nearly uniformly filled the terminal with 0.05 mmol/L EGTA (ratio = 0.88 ± 0.06, *n* = 16, *P* ≪ 0.000001). The pattern was similar in cones, with Ca^2+^ largely restricted to near the entry site with 5 mmol/L EGTA (ratio = 0.20 ± 0.04, *n* = 8) and spreading throughout the terminal when the EGTA concentration was lowered to 0.05 mmol/L (ratio = 0.81 ± 0.04, *n* = 6, *P *=* *0.00000064).

In rods, CICR boosts the average intraterminal Ca^2+^ concentration and extends the time course of Ca^2+^ signals (Krizaj et al. 2003; Cadetti et al. 2006; Babai et al. 2010b). While a portion of the enhanced Ca^2+^ signal observed with the 0.05 mmol/L EGTA pipette solution likely reflects the diminished buffering capacity, these conditions are also favorable for activation of CICR. To determine the contribution of CICR to the enhanced Ca^2+^ signals, we performed Ca^2+^ imaging while including the ryanodine receptor antagonist dantrolene (50 μmol/L) in the patch pipette (Fig. 3C and D). This reduced the amplitude of the Ca^2+^ signal and quickened the decay. With 100 msec steps, for instance, Δ*F*/*F*_0_ was reduced to 0.120 ± 0.011 (*n* = 6) in the presence of dantrolene (*P *=* *0.02). Additionally, the decay to 50% (*T*_50_) was 2.02 ± 0.31 sec (*n* = 7) with 0.05 mmol/L EGTA alone and 0.76 ± 0.22 sec (*n* = 7, *P *=* *0.014) when dantrolene was included. The spread of Ca^2+^ throughout the terminal was also significantly inhibited, with Ca_terminal_/Ca_hotspot_ being reduced to 0.56 ± 0.09 (*n* = 6, *P *= 0.016). Thus, a portion of the Ca^2+^ signal measured with 0.05 mmol/L EGTA reflects a boosting in amplitude, duration, and spatial extent of [Ca^2+^] due to enhanced CICR in rod terminals.

Previous studies with 5 mmol/L EGTA as the principal Ca^2+^ buffer in cones suggested that cone terminals do not exhibit CICR (Krizaj et al. 2003; Cadetti et al. 2006). We found that low endogenous-like buffering does not reveal a latent capability for CICR in cone terminals since, in contrast to rods, inclusion of dantrolene in the patch pipette had no effect on the Ca^2+^ signal (Fig. 4C and D). With a 100 msec step, the Δ*F*/*F*_0_ was 0.140 ± 0.016 (*n* = 6, *P *=* *0.6). Dantrolene also had no effect on the time course of the Ca^2+^ signal in cones; the *T*_50_ was 0.22 ± 0.02 sec (*n* = 6) with 0.05 mmol/L EGTA alone and 0.24 ± 0.02 sec (*n* = 8, *P* = 0.34) when dantrolene was included in the patch pipette. Likewise, Ca^2+^ spread was unchanged by dantrolene; Ca_terminal_/Ca_hotspot_ was 0.83 ± 0.06 (*n* = 8) with dantrolene, not significantly different from cones with 0.05 mmol/L EGTA alone (*P *=* *0.83). Thus, while endogenous-like Ca^2+^ buffering conditions enhance the amplitude and spread of Ca^2+^ in the cone terminal, this process does not involve CICR, in contrast to rods. Although ryanodine receptors are present in cone inner segments, strong Ca^2+^ extrusion mechanisms appear to prevent substantial CICR-evoked elevation in intraterminal [Ca^2+^] under most conditions (Krizaj et al. 2003, 2004). The slower decay of the Ca^2+^ signal in rods treated with dantrolene compared with cones is consistent with slightly weaker extrusion in rods (Krizaj et al. 2003; Van Hook and Thoreson 2014).

### Influences of endogenous-like Ca^2+^ buffering on synaptic transmission

**Figure 5:**
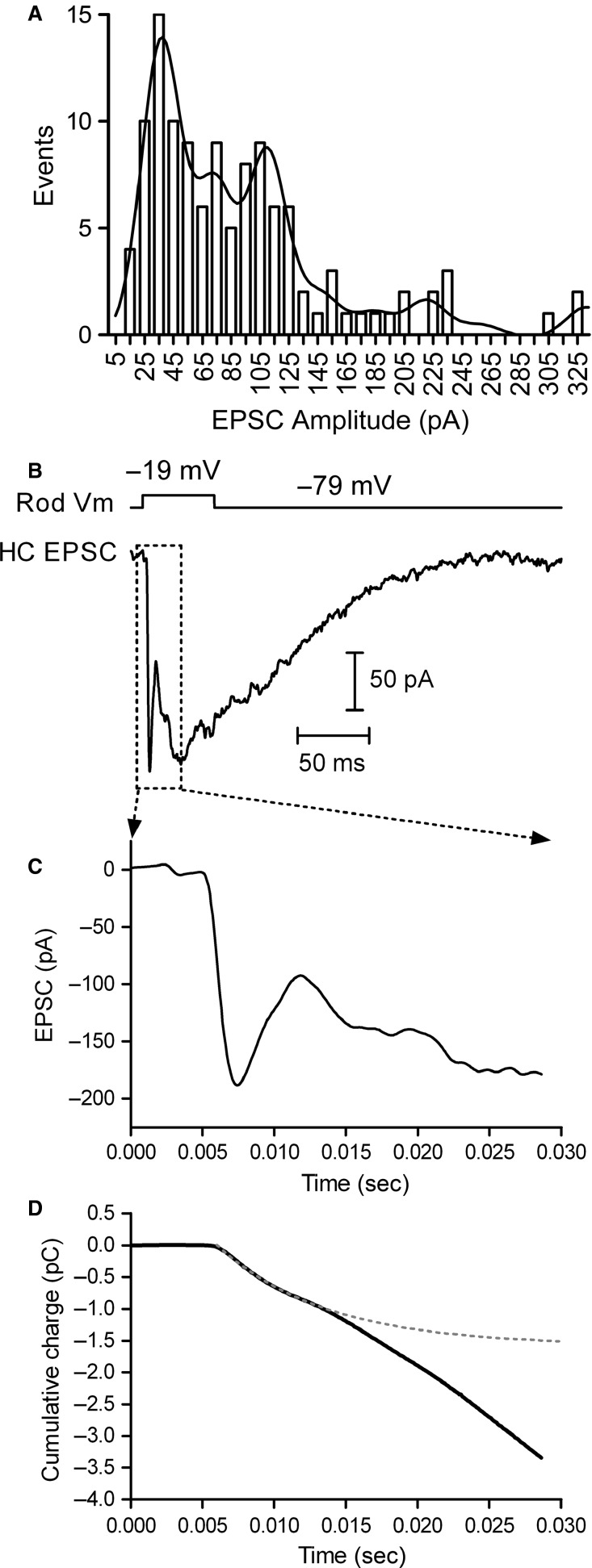
Immediately releasable pool (IRP) size in rods. (A) Distribution of rod-driven phasic EPSCs from 117 rod-HC pairs. The distribution was fit with a multiple Gaussian function with components of 35.9 pA, indicating that each ribbon contributes ˜36 pA to the rod-driven EPSC. The large peaks at 35.9, 71.8, and 107.7 pA indicate most rods make 1–3 ribbon contacts (average = 2.3) with each postsynaptic horizontal cell. (B–D) Analysis of the rod IRP from EPSC charge transfer measurements. (B) EPSC evoked by 50 msec depolarization to –19 mV in a rod-HC pair. The initial fast peak is the EPSC resulting from release of the IRP and had an amplitude of 188 pA, indicating that this rod made ˜5 ribbon contacts with the recorded horizontal cell. (C) Expanded view of the fast component of the EPSC. The expanded region is marked with the box in panel B. (D) Cumulative charge of the EPSC (black). The fast component, fit with a single exponential function (dotted gray), had a time constant of 7.5 msec and an amplitude of 1575 fC. Dividing by the number of ribbon contacts (5) and single vesicle charge (16 fC) gives a pool size of 19.7 vesicles/ribbon for this rod.

We next sought to determine how endogenous-like buffering conditions influence synaptic output of rod and cone photoreceptors using paired whole cell recordings of photoreceptors and postsynaptic horizontal cells. We began by characterizing properties of release at rod ribbons. Although previous work has quantitatively measured the IRP properties in cones (Bartoletti et al. 2010), a similar analysis has not been performed with rods. Therefore, we analyzed the amplitude of the fast rod-driven EPSCs recorded with rod-HC pairs to determine both the size of the ribbon-associated pool and the number of contacts each rod makes with each horizontal cell (Fig. 5). Most rod-HC pairs showed a combination of fast and slow components of the rod-driven EPSC, although a few exhibited only the fast component, similar to previous reports (Suryanarayanan and Slaughter 2006; Li et al. 2010). To measure the size of this fast ribbon-associated pool, we plotted a histogram of EPSC amplitudes from 117 rod-HC pairs, measuring the amplitude of the initial fast component at the beginning of each recording (Fig. 5A). The distribution could be described by a multiple Gaussian function with components of 35.9 pA, indicating that each ribbon contributed ˜36 pA to the fast, rod-driven EPSC, smaller than the 46 pA/ribbon component measured with a similar analysis of cone-driven responses (Bartoletti et al. 2010). The weighted average of these multiple Gaussian components suggests that each rod contacts a postsynaptic horizontal cell at an average of 2.3 ribbons. The smaller amplitude of the fast component in rods might be the result of fewer vesicles contributing to the rod-driven fast EPSC or might be the result of slower kinetics of release so that the same number of vesicles are released over a slightly longer time period. To test these possibilities, we measured the kinetics and amplitude of the first component of the cumulative charge transfer of the fast rod-driven EPSC with a single exponential function (Fig. 5B–D). This yielded a time constant of 9.2 ± 0.6 msec (*n* = 24), slower than measurements of IRP release from cones (6.1 ± 0.3 msec, *n* = 34, *P* = 0.00017) (Rabl et al. 2005; Bartoletti et al. 2010; Van Hook et al. 2014). When the amplitude of the fast component of EPSC charge transfer was scaled by the number of ribbon contacts measured from the EPSC peak amplitude (36 pA/ribbon) this gave a mean IRP amplitude of 383 ± 25 fC/ribbon (*n* = 24) or 25 ± 2 vesicles/ribbon (15.5 fC/vesicle) (Cadetti et al. 2008). Despite the fact that rod ribbons are believed to tether 6–7 times more vesicles than cone ribbons (Thoreson et al. 2004), this measurement of the rod IRP is only slightly larger than measurements of the cone IRP (˜15–20 vesicles/ribbon) (Bartoletti et al. 2010).

**Figure 6:**
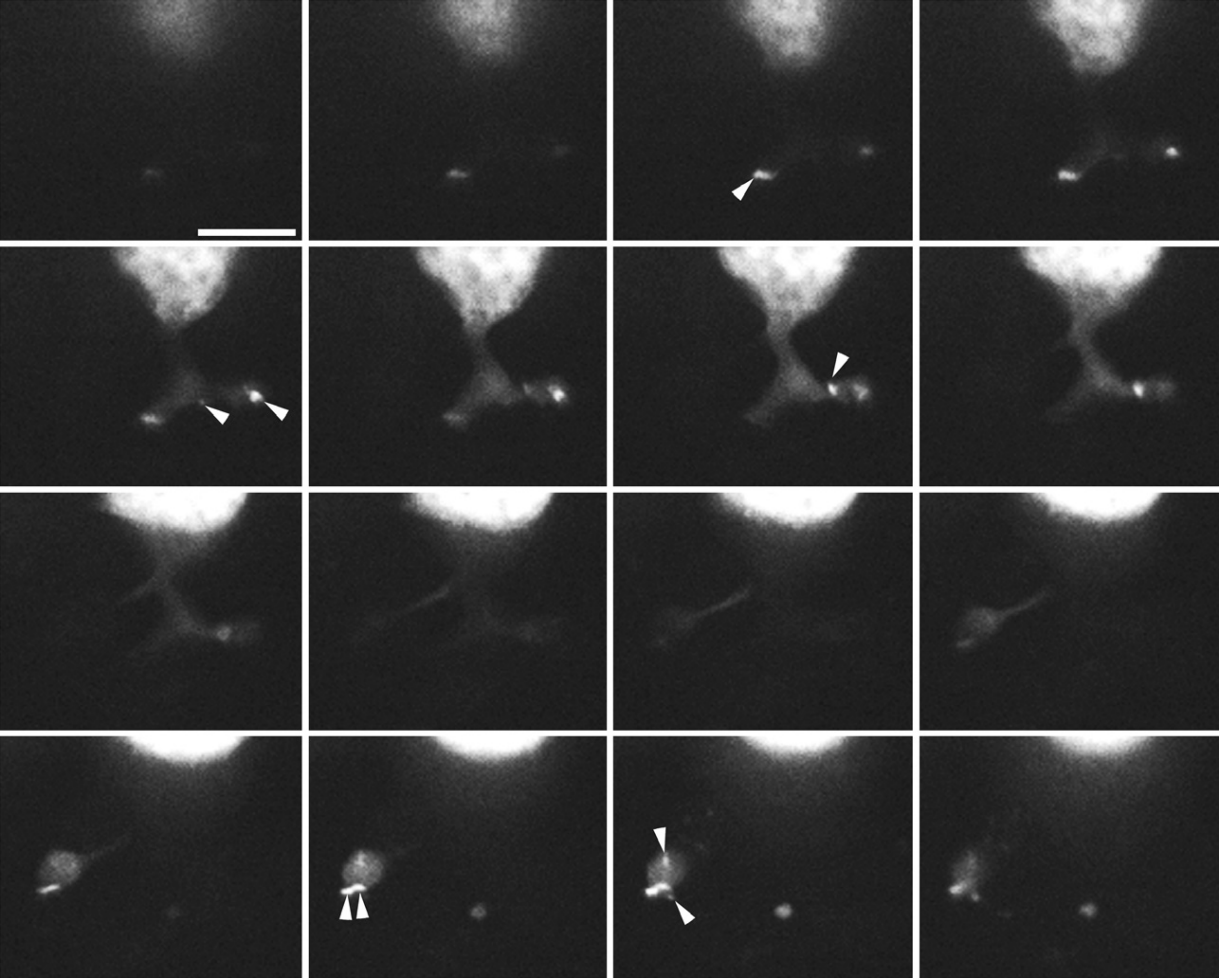
Synaptic ribbon number in rods. Sequential z-stack of images (1 *μ*m increments) through synaptic terminals of a rod dialyzed with the HiLyte488-Ribeye-binding peptide. Ribbons extend through multiple sections and were distinguished in three dimensions. Arrowheads mark HiLyte488 puncta corresponding to each of the eight ribbons visible in this rod. Scale = 10 *μ*m.

Previous studies have used electron microscopy to count synaptic ribbons in rods (Townes-Anderson et al. 1985; Pang et al. 2008). Because this parameter is key to performing an analysis of release efficiency, as we do below, we sought to verify these measurements using an independent experimental approach. To do this, we counted the number of ribbons in each rod using a HiLyte488-conjugated fluorescent Ribeye-binding peptide introduced through the patch pipette and imaging HiLyte488 puncta with a series of *z*-axis sections through rod terminals (Fig. 6). On average, each rod contained 6.6 ± 0.4 ribbons (*n* = 17 rods). This is lower than the number of ribbons counted in cone terminals using a similar approach (13 ribbons) (Bartoletti et al. 2010), but similar to counts of salamander rods using electron microscopy (Townes-Anderson et al. 1985). Salamander rods often have multiple synaptic terminals and Pang et al. (2008) counted 4.3 ribbons/terminal using electron microscopy. Counting HiLyte488 puncta in rod terminals, we obtained a similar value of 4.1 ± 0.4 ribbons/terminal (*n* = 27 terminals). This contrasts with spherules in fish and mammals, which commonly have only a single large synaptic ribbon (Van Haesendonck and Missotten 1984; Hidaka et al. 1986; Rao-Mirotznik et al. 1995; Zampighi et al. 2011).

**Figure 7:**
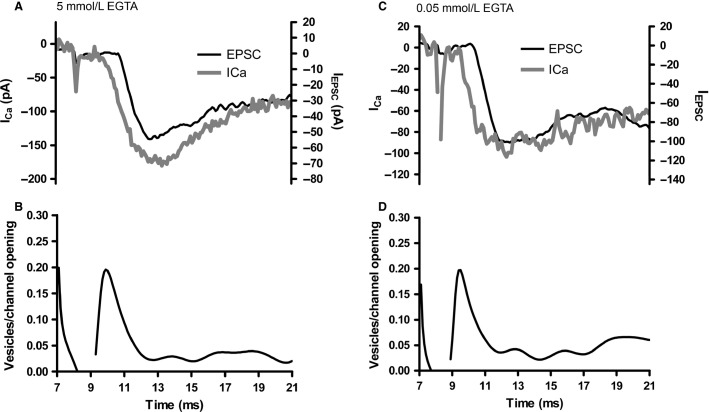
Effects of Ca^2+^ buffering on rod release efficiency. (A) Rod Ca^2+^ current (*I*_C__a_, gray, left axis) and overlaid EPSCs (black, right axis) in response to a depolarizing step (–79 to –19 mV, 100 msec). The rod pipette solution contained 5 mmol/L EGTA and *I*_C__a_ was recorded with a P/8 leak subtraction protocol. The inward current deflection at ˜8 msec is an artifact from incompletely compensated pipette capacitance and did not influence the efficiency measurement. (B) The number of vesicle fusion events per Ca^2+^ channel opening measured by deconvolving both the EPSC and *I*_C__a_. The peak efficiency for this recording was 0.2 vesicles/channel opening. (C) Overlaid EPSC and *I*_C__a_ recorded from a rod-HC cell pair in which the rod patch pipette solution contained 0.05 mmol/L EGTA to mimic endogenous Ca^2+^ buffering. (D) The peak efficiency for this recording was 0.2 vesicles/channel opening, similar to the 5 mmol/L EGTA recording.

In cones, deconvolution analysis of voltage-gated Ca^2+^ currents and simultaneously recorded EPSCs indicates that a single vesicle fusion event can be triggered by ≤3 Ca^2+^ channel openings (Bartoletti et al. 2011). We performed a similar analysis in rods (Fig. 7), measuring the release efficiency (number of vesicle fusion events per Ca^2+^ channel opening) of the fast component of the rod-driven EPSC when the rod pipette solution contained either 5 or 0.05 mmol/L EGTA. Based on the measurements of rod ribbon number above, we calculated the contribution of Ca^2+^ channel openings at each ribbon by scaling the Ca^2+^ current by seven ribbons per rod. We also scaled the EPSC amplitude by assuming that each ribbon contributed 36 pA to the fast component of the EPSC recorded in each horizontal cell, based on the multiple Gaussian fit of the rod-driven EPSC amplitude histogram. With 5 mmol/L EGTA, efficiency of release at rod synapses averaged 0.17 ± 0.03 vesicles/channel opening, or approximately six channel openings per vesicle fusion event (*n* = 16 rod-HC pairs). When we instead dialyzed the rod with 0.05 mmol/L EGTA to mimic the endogenous buffering conditions, efficiency was 0.21 ± 0.3 vesicles/channel opening, or ˜5 channel openings per vesicle fusion (*n* = 11). Although release efficiency was slightly higher with 0.05 mmol/L EGTA than 5 mmol/L EGTA, the difference was not significant (*P *=* *0.33), suggesting that this 100-fold change in Ca^2+^ buffer concentration does little to alter Ca^2+^ domains responsible for triggering fast exocytosis.

**Figure 8:**
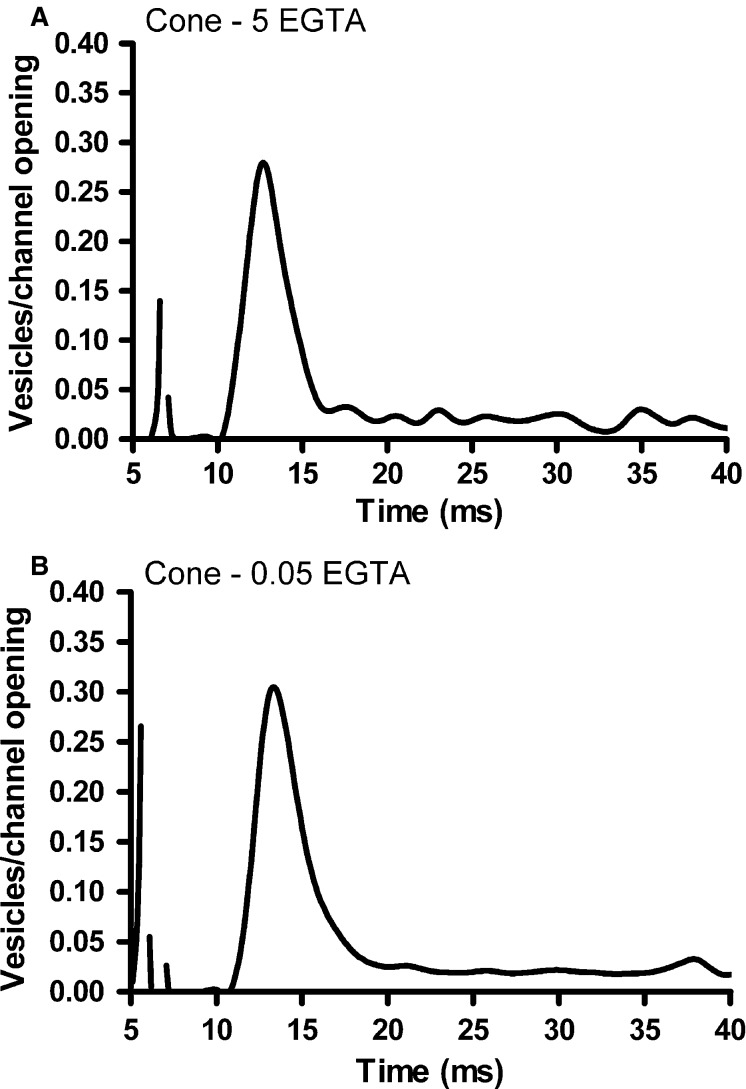
Effects of Ca^2+^ buffering on cone release efficiency. (A) Cone release efficiency (number of vesicles/channel opening), measured by deconvolution of the EPSC and cone *I*_C__a_ evoked with a 100 msec step to –19 mV from a holding potential of –79 mV in a cone-horizontal cell pair in which the cone pipette solution contained 5 mmol/L EGTA, similar to Figure 6. (B) Peak efficiency was similar in another cone-HC pair when the cone pipette solution contained 0.05 mmol/L EGTA to mimic endogenous Ca^2+^ buffering.

We performed a similar analysis of release efficiency with cones (Fig. 8). With 5 mmol/L EGTA, cone release efficiency was 0.25 ± 0.02 vesicles/channel opening (*n* = 15 cone-HC pairs), or ˜4 channel openings per fusion event, similar to previous measurements in cones (Bartoletti et al. 2011). Additionally, the concentration of exogenous buffer had no significant effect on release efficiency in cones, which was 0.27 ± 0.01 vesicles/channel (*n* = 23; *P *=* *0.84) when the EGTA concentration was reduced to 0.05 mmol/L to mimic endogenous buffering. This agrees with previous results, where efficiency was unchanged whether the cone was recorded under perforated patch configuration or dialyzed with exogenous Ca^2+^ buffers (Bartoletti et al. 2011). When we pooled across buffering conditions, the values of release efficiency in cone-HC pairs were significantly higher than the measurements made with rod-HC pairs (mean rod efficiency = 0.19 ± 0.02, mean cone efficiency = 0.26 ± 0.01, *P *=* *0.007).

**Figure 9:**
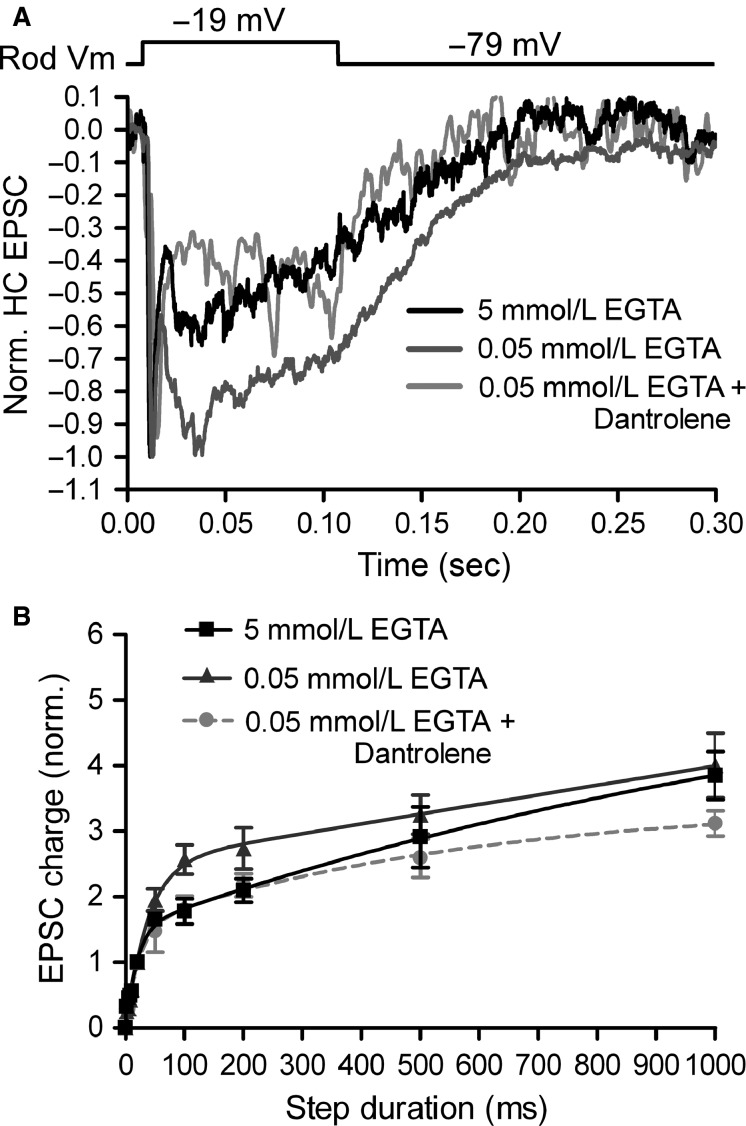
Low Ca^2+^ buffering enhances CICR-mediated sustained release from rods. (A) Rod-driven EPSCs, normalized to the peak of the fast phasic component, recorded in response to a 100 msec depolarizing step (–79 to –19 mV) in rod-HC pairs in which the rod pipette contained 5 mmol/L EGTA (black), 0.05 mmol/L EGTA (dark gray), or 0.05 mmol/L EGTA with 10 μmol/L dantrolene in the bath solution (light gray). (B) Group data of normalized EPSC charge showing that release was enhanced at intermediate stimulus durations when the rod pipette contained 0.05 mmol/L EGTA. With 0.05 mmol/L EGTA, release was inhibited at longer durations when 10 μmol/L dantrolene was bath applied to the slices.

We next probed the influence of 0.05 mmol/L EGTA on rod release kinetics by stimulating rods with depolarizing steps of varying duration (2–1000 msec) while simultaneously recording postsynaptic responses from voltage-clamped horizontal cells (Fig. 9). We minimized the contribution of release from electrically coupled neighboring photoreceptors by applying a strong background illumination while recording each response. In these experiments, a brief depolarizing stimulus triggered a fast EPSC that decayed quickly back to baseline. And, as the duration of the stimulus increased, slower components of the rod-driven EPSC began to emerge. Although the shape of these components and their amplitude relative to the fast component were variable from cell to cell, plotting mean EPSC charge against stimulus duration revealed that postsynaptic responses were enhanced, especially at intermediate stimulus durations, when the pipette solution contained 0.05 mmol/L EGTA. To confirm that the different buffering conditions did not alter postsynaptic responses by altering rod–rod electrical synapses (component #3 in Fig. 1B), we directly measured the gap junctional conductance (G_j_) between adjacent rods with paired whole cell recordings. In these experiments, the G_j_ was 1228 ± 1188 pS with 5 mmol/L EGTA (*n* = 7 rod–rod pairs) and 1206 ± 196 pS with 0.05 mmol/L EGTA (*n* = 8 rod–rod pairs, *P *=* *0.94). These values are more than double the previously reported rod–rod G_j_ in salamander retinal slices (˜500 pS) (Zhang and Wu 2005), a difference that could be the result of different lighting conditions in our experiments. Regardless, the similarity of G_j_ with 5 and 0.05 mmol/L EGTA in our experiments indicate that the buffering levels did not alter electrical coupling and that the enhanced postsynaptic currents measured with the 0.05 mmol/L EGTA were not the result of enhanced release from gap junctionally coupled neighboring rods.

As Ca^2+^-imaging experiments revealed that CICR activation in rods was favored with 0.05 mmol/L EGTA, we tested the contribution of CICR to this enhanced synaptic output by bath-applying 10 μmol/L dantrolene. Dantrolene has been shown to block CICR in rods (Chen et al. 2014) and, consistent with a role for CICR in promoting sustained release (Cadetti et al. 2006), it caused a reduction in release with intermediate and long stimulus durations. The addition of dantrolene only affected EPSCs evoked by longer duration stimuli, as the responses were nearly identical for stimuli <50 msec (*P* > 0.05). With 5 mmol/L EGTA, in contrast, the responses began to diverge from the dantrolene-treated condition only with very long stimuli (>500 msec), suggesting that the strong buffering prevented average intraterminal [Ca^2+^] from reaching sufficiently high concentrations to trigger CICR and nonribbon release. These data indicate that introducing exogenous Ca^2+^ buffers designed to mimic the endogenous conditions boosts sustained components of synaptic transmission by enhancing CICR in rods.

**Figure 10:**
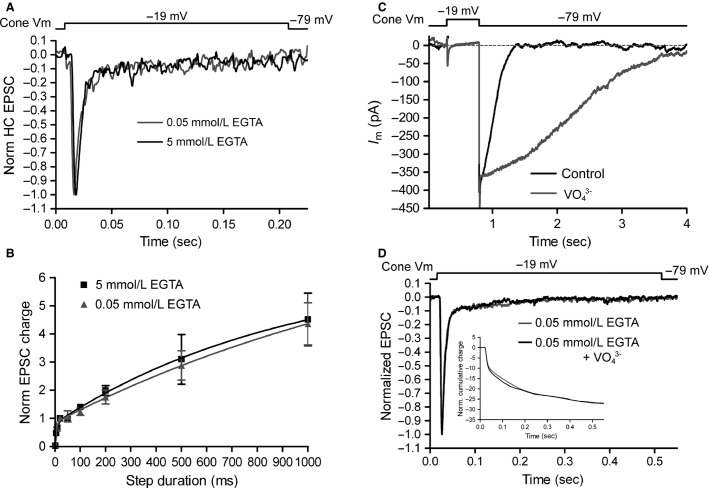
Low Ca^2+^ buffering does not trigger nonribbon release in cones. (A) Cone-driven EPSCs (normalized to the peak) recorded in horizontal cells in response to 200 msec steps (–79 to –19 mV). The kinetics are similar, suggesting that reduced Ca^2+^ buffering does not recruit additional components of release over this time scale. (B) Group data of normalized charge of cone-driven EPSCs in response to stimuli of varying duration (1–1000 msec) when the cone pipette solution contained either 0.05 (gray) or 5 mmol/L (black) EGTA. (C) The duration of *I*_C__l(Ca)_, evoked with a 500 msec voltage step (–79 mV to –19 mV) in cones is prolonged when cones were dialyzed with 1 mmol/L intracellular orthovanadate (VO_4_^3–^), indicating that VO_4_^3–^ extends the duration of elevated [Ca^2+^] in the terminal. (D) The kinetics of normalized cone-driven EPSCs from two different cone-HC pairs (0.05 mmol/L EGTA in cone pipettes) were unchanged when Ca^2+^ extrusion was inhibited with 1 mmol/L intracellular VO_4_^3–^. Inset: Cumulative charge transfer showing that release kinetics were similar in the absence and presence of VO_4_^3–^.

In contrast to rods, which rely heavily on nonribbon release, it has been suggested that all of the exocytosis in cones involves the synaptic ribbon (Snellman et al. 2011). With Ca^2+^ imaging experiments above, we showed that CICR does not contribute to the depolarization-induced elevation of intraterminal Ca^2+^ concentration in cones. However, in bipolar cells that also do not exhibit CICR, nonribbon release can nevertheless be revealed after saturating intracellular Ca^2+^ buffering (Mehta et al. 2014). Are cones also capable of exocytosis at nonribbon sites when using weak endogenous Ca^2+^ buffering that promotes buffer saturation and elevated intraterminal [Ca^2+^] levels? We tested this by stimulating exocytosis from cones with a depolarizing step (to –19 mV from a holding potential of –79 mV) with durations of 2–1000 msec while simultaneously recording postsynaptic responses in voltage-clamped horizontal cells (Fig. 10). Although the Ca^2+^ imaging studies shown in Figure 4 confirmed that low buffering allowed [Ca^2+^] to reach high levels throughout the terminal, we did not observe the emergence of any additional components of release when the cone patch pipette contained 0.05 mmol/L EGTA; the postsynaptic responses were nearly identical in both buffering conditions when cones were stimulated with pulses with durations ranging from 2 to 1000 msec (Fig. 10B).

In the inner segments of some cones, inhibition of the plasma membrane Ca^2+^ ATPase (PMCA) was found to unmask strong caffeine-evoked Ca^2+^ signals suggesting that elevation of Ca^2+^ within the cone synaptic terminal due to CICR can be counterbalanced by strong Ca^2+^ extrusion and sequestration (Krizaj et al. 2003). We therefore tested whether slower, possibly nonribbon components of cone exocytosis might be revealed when the pipette solution contained 0.05 mmol/L EGTA and PMCA was inhibited by orthovanadate (1 mmol/L, included in the pipette solution) (Morgans et al. 1998). We first verified that the orthovanadate pipette solution extended the time course of Ca^2+^ signals by recording *I*_Cl(Ca)_ from cones. Using a CsCl-based pipette solution (*E*_Cl_ = –2.7 mV) to enhance *I*_Cl(Ca)_ at the holding potential by increasing Cl^–^ driving force, the inclusion of orthovanadate slowed the time to 50% recovery (*t*_50_) of the *I*_Cl(Ca)_ following 1 sec steps to –19 mV from 191 ± 27 msec (*n* = 9) to 639 ± 166 msec (*n* = 7, *P* < 0.05) indicating that inhibition of extrusion dramatically enhances the duration of Ca^2+^ signals in cones (Fig. 10C). Despite this enhancement, however, in paired recordings with the Cs-glutamate/Cs-gluconate and 0.05 mmol/L EGTA presynaptic pipette solution, inclusion of orthovanadate had no effect on the kinetics of cone-driven EPSCs evoked by 500 msec steps (Fig. 10D, *n* = 4 control cells, *n* = 3 cells with orthovanadate). This indicates that, in contrast to rods (Chen et al. 2013, 2014), cones do not appear to be capable of ectopic, nonribbon release even with high levels of Ca^2+^ throughout the entire terminal.

Replenishment of vesicles to the synaptic ribbon in cones is accelerated by Ca^2+^ in a process that involves actions of CaM on ribbon-associated proteins to enhance vesicle attachment (Babai et al. 2010b; Van Hook et al. 2014). Endogenous levels of Ca^2+^ buffering might be able to support sustained synaptic transmission by cones by enhancing Ca^2+^/CaM-dependent acceleration of replenishment. To test this possibility, we measured exocytosis from cones under conditions where the rate of release was limited by the rate of replenishment (Fig. 1). Cones were voltage clamped at –79 mV and stimulated with a train of depolarizing pulses (13.3 Hz) to –39 mV for 2 sec followed by –10 mV for 2 sec. In this stimulus paradigm, the slope of a linear increase in cumulative charge transfer of the postsynaptic response represents the rate of replenishment (Sakaba et al. 2002; Babai et al. 2010a; Van Hook et al. 2014). With the transition from –39 to –19 mV, the slope of the cumulative charge transfer steepens, indicative of enhanced CaM-dependent replenishment from increased Ca^2+^ influx (Babai et al. 2010a; Van Hook et al. 2014). When the cone patch pipette contained 5 mmol/L EGTA, this slope increased by a factor of 1.57 ± 0.15 with the transition from –39 mV to –19 mV (*n* = 9). When the cone pipette solution instead contained 0.05 mmol/L EGTA, the change in the slope was significantly greater than with 5 mmol/L EGTA, increasing by a factor of 2.59 ± 0.21 (*n* = 17, *P *=* *0.00055). When the cone pipette contained 0.05 mmol/L EGTA and slices were treated with the CaM inhibitor calmidazolium (20 μmol/L), the slope only increased by a factor of 1.28 ± 0.06 (*n* = 7, *P *=* *0.0000084).

**Figure 11:**
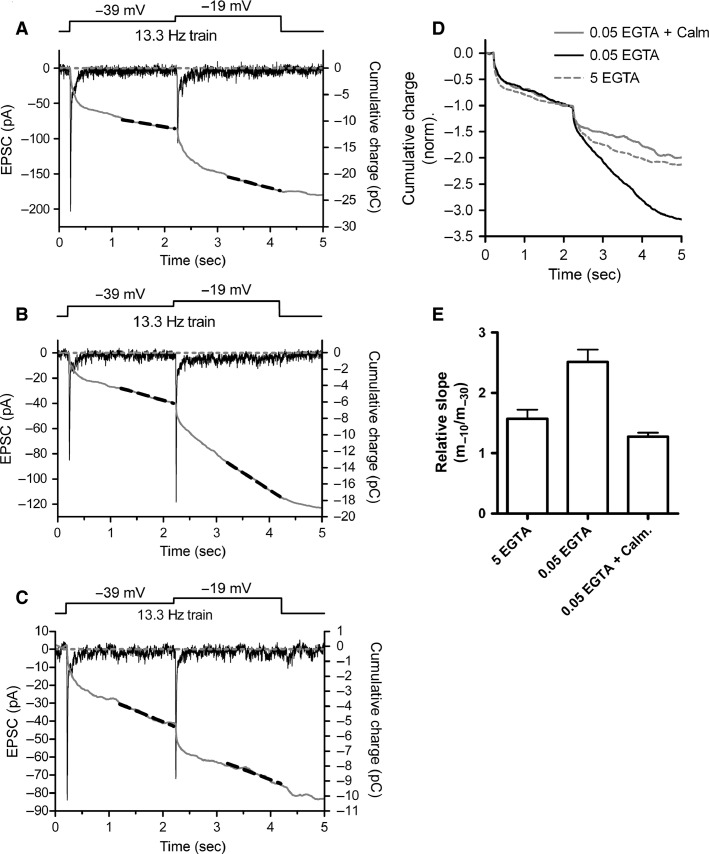
Reduced Ca^2+^ buffering promotes Ca^2+^/calmodulin-dependent acceleration of replenishment in cones. (A) Paired cone-HC recording in which the cone was stimulated with a train of depolarizing pulses (25 msec duration, 13.3 Hz) to –39 mV for 2 sec followed by –19 mV for 2 sec (*V*_hold_ = –79 mV). The black trace is the postsynaptic current (left axis). The dashed horizontal gray line is the prestimulus baseline. The gray trace is the cumulative charge of the EPSC (right axis). Baseline synaptic input was subtracted by adjusting the slope of the prestimulus baseline. Replenishment rate was measured as the slope of a linear fit (black dashed lines) to the last 1 sec at –39 and –19 mV. Under these conditions, in which release is limited by the rate of replenishment, the increase in slope represents an increase in replenishment rate. (B) A similar recording in which the cone pipette solution contained 0.05 mmol/L EGTA to mimic endogenous buffering conditions. Under these conditions, the change in slope measured with the transition from –39 to –19 mV was greater, indicating that 0.05 mmol/L EGTA enhances replenishment rate. (C) Bath application of the calmodulin inhibitor calmidazolium (20 μmol/L) reduced the change in slope accompanying the transition from –39 mV to –19 mV. (D) EPSC cumulative charge traces from A to C normalized to the 2 sec time point to emphasize the differences in the relative slope under different experimental conditions. (E) Group data of relative slope (slope_–10_/slope_–30_) showing that the increase in replenishment rate was enhanced when the cone pipette contained 0.05 mmol/L EGTA. This enhancement from 0.05 mmol/L EGTA was inhibited when the retinal slices were treated with 20 μmol/L calmidazolium.

We next tested whether CaM can also regulate the replenishment of vesicles to rod synaptic ribbons (Fig. 2). Because the large and slow nonribbon components in the rod-driven EPSC make cumulative charge transfer measurements of ribbon replenishment impractical, we used a paired pulse protocol, stimulating rods with pairs of depolarizing steps (100 msec steps from –79 to –19 mV) at intervals of 0.5, 2, and 10 sec while measuring the paired pulse ratio (PPR) of the fast, ribbon-mediated component of the EPSC. Because AMPA receptor desensitization makes little contribution to the recovery from synaptic depression over these time scales (Rabl et al. 2006; Van Hook et al. 2014), this process represents the replenishment of release-ready vesicles to the rod synaptic ribbon. In the presence of calmidazolium (20 μmol/L, bath applied), the PPR was reduced at both the 2 sec interval (control PPR = 0.56 ± 0.07, *n* = 13; calmidazolium PPR = 0.38 ± 0.04, *n* = 10; *P *<* *0.05) and the 10 sec interval (control PPR = 1.06 ± 0.05, *n* = 11; calmidazolium PPR = 0.89 ± 0.05, *n* = 10; *P *<* *0.05). Synaptic transmission was strongly depressed at the 500 msec interval and was not significantly different between control and calmidazolium-treated conditions (control PPR = 0.046 ± 0.030, *n* = 6; calmidazolium PPR = 0.016 ± 0.019, *n* = 6; *P *>* *0.05). This appears somewhat slower than similar measurements in cones (Van Hook et al. 2014) and considerably slower than in hair cells (Cho et al. 2011), which also possess a synaptic ribbon, possibly reflecting the different temporal signaling requirements of each synapse. These data suggest that low Ca^2+^ buffering enhances sustained release in cones exclusively through CaM-dependent replenishment to the ribbon, whereas sustained release from rods is boosted by a combination of CaM-mediated ribbon replenishment and nonribbon CICR-driven exocytosis.

**Figure 12:**
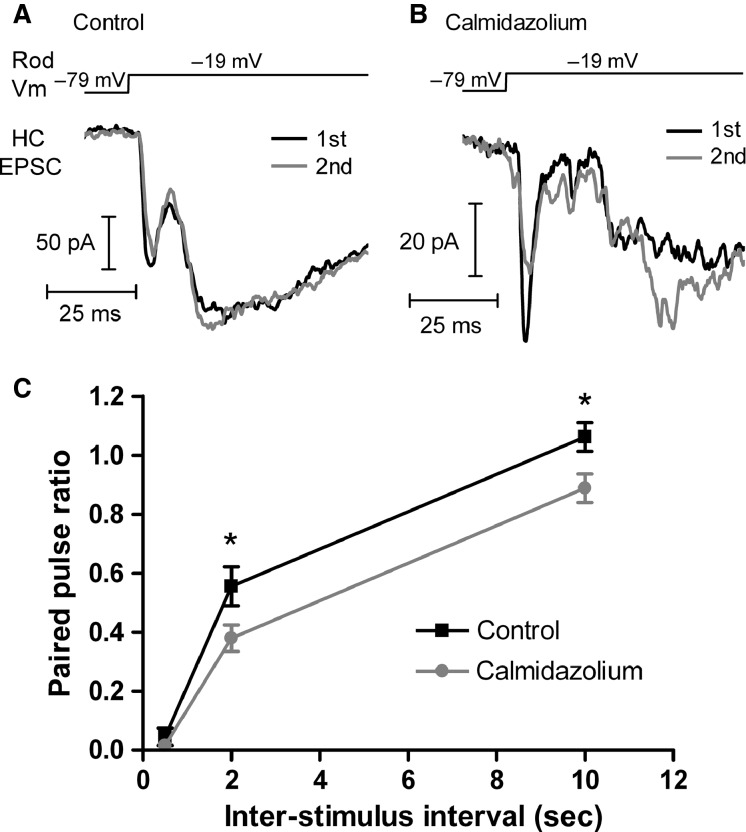
Calmodulin regulates vesicle replenishment of vesicles to the rod synaptic ribbon. (A) Paired rod-HC recording in which the rod was depolarized with a pair of voltage steps from –79 to –19 mV with an interval of 10 sec between steps. The fast, ribbon-mediated component of the EPSC recovered nearly 100% by the second pulse. (B) In a rod-HC pair treated with calmidazolium (20 μmol/L) and stimulated as in panel A, the recovery of the fast component of the EPSC was slowed. (C) Group data showing the paired pulse ratio (EPSC_2_/EPSC_1_) for pairs of depolarizing pulses with intervals of 0.5, 2, and 10 sec. *P <* *0.05.

## Discussion

This study shows that, aside from a slightly lower release efficiency in rods (i.e., the number of Ca^2+^ channel openings required to trigger fusion of a single vesicle), the properties of fast ribbon-mediated release are generally similar in rods and cones. Fast phasic transmission from rods and cones was also not significantly altered by conditions that mimic native Ca^2+^ buffering in the synaptic terminal, which is equivalent to ˜0.05 mmol/L EGTA (Van Hook and Thoreson 2014). At bipolar cell synapses, another ribbon-type retinal synapse, a variety of measurement approaches have indicated that the endogenous buffering is considerably stronger, equivalent to approximately 1–2 mmol/L BAPTA (Burrone et al. 2002; Singer and Diamond 2003; Mehta et al. 2014). Similar values have also been reported for hair cells (Frank et al. 2009; Pangršič et al. 2015). While it did not alter fast transmission, the use of weak endogenous Ca^2+^ buffering in photoreceptors enhanced slow, sustained synaptic transmission. In cones, this enhancement of sustained release is due to the ability of Ca^2+^/CaM to speed replenishment of vesicles to the ribbon. We found that rods share a similar mechanism of Ca^2+^/CaM-stimulated vesicle replenishment. In addition, rods but not cones exhibit a specialized capability for substantial release at nonribbon sites triggered by CICR. This mechanism is also enhanced by low endogenous Ca^2+^ buffering. Low endogenous buffering may thus be an adaptation of photoreceptors to promote sustained release under dim light conditions, acting at ribbons in cones and at both ribbons and nonribbon sites in rods.

### Rod synaptic vesicle pools

In both rods and cones, the initial fast component of the depolarization-evoked EPSC involves release from synaptic ribbons and is therefore reduced by damage to ribbons (Chen et al. 2013, 2014). We characterized the properties of ribbon release from rods to determine whether they are similar to those of cones (Bartoletti et al. 2010). We found that although the peak amplitude of the rod-driven fast EPSC associated with each ribbon was slightly smaller (˜36 pA/ribbon) than in cones (˜47 pA/ribbon, Bartoletti et al. 2010), the pool size in rods was slightly larger (˜25 vesicles/rod ribbon compared to 15–20 vesicles/cone ribbon). The smaller amplitude reflects slightly slower release kinetics in rods, leading to slightly less synchronized release of vesicles in the IRP. In salamander cones, the ribbon appears to tether ˜110 vesicles with the bottom two rows (15–20 vesicles) in contact with the presynaptic membrane (Lasansky 1973, 1978; Jackman et al. 2009). These bottom two rows appear to comprise the IRP (Jackman et al. 2009; Bartoletti et al. 2010), which, under physiological conditions, is depleted during a transition to darkness following a bright flash, as simulated by our depolarizing voltage steps. This process is a key for efficiently encoding contrast at the cone synapse (Jackman et al. 2009; Bartoletti et al. 2011). Ultrastructural measurements indicate that salamander rod ribbons are larger than cone ribbons and can tether ˜710 vesicles/ribbon (Lasansky 1973, 1978; Thoreson et al. 2004; Pang et al. 2008), but only ˜25 vesicles/ribbon contact the adjacent plasma membrane (Heidelberger et al. 2005), consistent with the IRP measurement of 25 vesicles/ribbon. The number of vesicles released in response to a given stimulus is determined by both release probability (*Pr*) and pool size (*N*) (Del Castillo and Katz 1954) and changes in Ca^2+^ influx can regulate both *Pr* and *N* (Thanawala and Regehr 2013). If rod IRP size is determined in part by the distance of vesicles from the Ca^2+^ channels, further increases or decreases in Ca^2+^ influx via modulatory effects of dopamine, adenosine, insulin, somatostatin, or endocannabinoids might serve to enhance or diminish rod output in part by regulating effective IRP size (Akopian et al. 2000; Stella and Thoreson 2000; Stella et al. 2002; Thoreson et al. 2002; Straiker and Sullivan 2003).

### Phasic release efficiency

Fast phasic release in both rods and cones was unaffected by the 100-fold difference in Ca^2+^ buffer concentrations used in this study. Release efficiency in cones has previously been shown to be unaffected by modest changes in Ca^2+^ buffering or when endogenous buffers were kept intact in perforated patch recordings (Bartoletti et al. 2011). Even use of 1 mmol/L BAPTA had no effect on cone release efficiency (Bartoletti et al. 2011), although greater concentrations (i.e., 5–10 mmol/L BAPTA) inhibited the release (Mercer et al. 2011; Van Hook and Thoreson 2012). The relative insensitivity of exocytosis to high [EGTA] and only low sensitivity to BAPTA is consistent with nanodomain coupling (<100 nm) of Ca^2+^ channels to release machinery (Adler et al. 1991; Mercer et al. 2011; Eggermann et al. 2012) in cones. Ribbon-bearing hair cells and retinal bipolar cells also appear to rely on nanodomain coupling (Brandt et al. 2005; Jarsky et al. 2010), although some work does point toward microdomain coupling in bipolar cells (Coggins and Zenisek 2009).

In cones, Ca^2+^ nanodomains are created by the gating of 3–4 Ca^2+^ channels to trigger fusion of a single synaptic vesicle (Bartoletti et al. 2011). Some estimates indicate that only ˜70% of Ca^2+^ channels are located at the photoreceptor synapse (Steele et al. 2005; Szikra and Krizaj 2006) and so as few as 1–2 channels might be sufficient to trigger release in cones (Bartoletti et al. 2011). This phasic release process is likely to play a role in bright light conditions, when cones depolarize abruptly at the cessation of a bright flash, as simulated with our voltage-clamp stimuli. In rods, fusion of a single vesicle from the ribbon-associated fast pool required ˜5–6 Ca^2+^ channel openings, slightly less efficient than in cones. Estimates indicate that ˜95% of rod Ca^2+^ channels are in the rod synaptic terminal (Xu and Slaughter 2005), although if, as in hair cells, not all are located at the synaptic ribbon (˜70% and ˜50% in mature and immature hair cells, respectively) (Brandt et al. 2005; Zampini et al. 2010), then each vesicle fusion event in rods could be triggered by as few as 3–4 Ca^2+^ channel openings. In cones, release efficiency is strongly influenced by the presence of a diffusion barrier, likely the arciform density (Bartoletti et al. 2011). Because the Ca^2+^ affinity of the release sensor is similarly high in rods and cones (Duncan et al. 2010), the small differences in efficiency between the two photoreceptor types could be due a combination of slightly farther Ca^2+^ channel vesicle diffusion distance and/or to other subtle differences in synaptic architecture such as positions or size of the ribbon and arciform density.

Nanodomain control of exocytosis is typically associated with fast synchronized neurotransmitter release (Brandt et al. 2005; Bucurenciu et al. 2010; Eggermann et al. 2012) and so the slightly lower rod release efficiency is therefore likely to contribute to the slightly slower rod IRP release kinetics relative to cones (Schnapf and Copenhagen 1982; Copenhagen et al. 1983; Cadetti et al. 2005; Rabl et al. 2005). The speed and efficiency of nanodomain-triggered exocytosis is key for temporally precise spike generation in the auditory system (Brandt et al. 2005; Fedchyshyn and Wang 2005) and allows for linear encoding of information over a wide dynamic range in the visual system (Jackman et al. 2009; Jarsky et al. 2010; Oesch and Diamond 2011).

### Ca^2+^/CaM-dependent acceleration of replenishment

Cones release glutamate in three kinetically distinct phases. The first represents fast, highly efficient exocytosis of the immediately releasable pool of vesicles from the base of the ribbon (˜15–20 vesicles, *τ *= 2–6 msec). A second, slower phase is the result of the ribbon-associated reserve pool moving down the ribbon and being released with *τ *= 100–500 msec (Rabl et al. 2005; Bartoletti et al. 2010). A third, linear component is the result of ongoing release being rate limited by the replenishment of vesicles to the ribbon (Babai et al. 2010a; Bartoletti et al. 2010). The rate of this third phase can be accelerated by manipulations that boost the intraterminal Ca^2+^ concentration (Babai et al. 2010a; Van Hook et al. 2014). A combination of experimental and modeling work indicates that this is the result of CaM enhancing a fast replenishment process (*τ* ˜ 800 msec) by acting on synaptic ribbon sites to increase the probability that a colliding vesicle will attach (Van Hook et al. 2014). In this study, we found that mimicking low endogenous buffering conditions with 0.05 mmol/L EGTA enhanced Ca^2+^/CaM-dependent replenishment in cones. We also found that inhibition of CaM slowed recovery from synaptic depression in rods, showing that rod ribbons also possess similar mechanisms for Ca^2+^/CaM-dependent acceleration of replenishment and sustained release.

### Differential influences of endogenous Ca^2+^ buffering on rod and cone synaptic transmission

Rod and cones differ dramatically in their release kinetics (Pasino and Marchiafava 1976; Baylor and Fettiplace 1977; Schnapf and Copenhagen 1982; Copenhagen et al. 1983; Witkovsky and Stone 1983; Cadetti et al. 2005; Rabl et al. 2005; Thoreson 2007). While both rods and cones release vesicles at the synaptic ribbon, a substantial portion of rod release is the result of CICR-driven exocytosis at nonribbon sites (Chen et al. 2013, 2014). This slow CICR-driven nonribbon release accounts for the overall slower release kinetics in rods and makes a substantial contribution (>50%) to the light responses of downstream retinal neurons (Krizaj et al. 1999; Cadetti et al. 2006; Suryanarayanan and Slaughter 2006; Babai et al. 2010b). Using Ca^2+^ imaging of rod and cone synaptic terminals, we found that endogenous levels of buffering allowed for larger and more sustained Ca^2+^ signals. In rods but not cones, this was due, in part, to enhanced CICR. This led to increased exocytosis during steps of intermediate duration (50–500 msec), consistent with a greater proportion of Ca^2+^ ions contributing to free [Ca^2+^] and thereby triggering CICR-mediated nonribbon exocytosis during shorter stimuli (Cadetti et al. 2006; Suryanarayanan and Slaughter 2006; Chen et al. 2013, 2014).

### Nonribbon sustained release in cones?

One key question we sought to address in this study was whether cones are capable of nonribbon sustained synaptic transmission. Previous work has shown that CICR generally does not contribute to intraterminal Ca^2+^ in cones and that cone-driven EPSCs rely entirely on the ribbon (Krizaj et al. 2003; Snellman et al. 2011). Even when using low endogenous Ca^2+^ buffering, we did not observe evidence of CICR in cones. However, retinal bipolar cells (BCs) which do not exhibit CICR are nevertheless capable of sustained nonribbon release triggered by high [Ca^2+^] following buffer saturation (Zenisek et al. 2002; Midorikawa et al. 2007; Zenisek 2008; Mehta et al. 2014). When we used paired recordings to measure postsynaptic responses, low endogenous levels of Ca^2+^ buffering and the resultant enhancement of [Ca^2+^] in the cone terminal did not recruit additional slow components of the postsynaptic response, even when PMCA-dependent extrusion was dramatically inhibited. Thus, unlike rods or bipolar cells, cones do not appear to be capable of employing nonribbon release.

An alternative interpretation of these results is that nonribbon release may occur in cones but that it went undetected in our experiments. In hair cells, reducing intracellular Ca^2+^ buffering by knocking out key Ca^2+^ binding proteins enhances exocytosis measured with capacitance recordings, but has no detectable effects downstream in the auditory pathway (Pangršič et al. 2015). This indicates that although reduced Ca^2+^ buffering enhances sustained release in hair cells, the additional glutamate was not detected postsynaptically. It is unlikely, however, that nonribbon release by cones would go similarly undetected. In the retina, residual levels of cleft glutamate derived from rod release can make a substantial contribution to horizontal and bipolar cell responses (Cadetti et al. 2008) and glutamate released by one cone can evoke sizeable glutamate transporter currents in neighboring cones (Szmajda and Devries 2011; Vroman and Kamermans 2015), indicating that excess cleft glutamate, as would be produced by nonribbon release, would diffuse far enough and reach a sufficiently high concentration to contribute to horizontal cell responses.

The involvement of CICR-driven nonribbon release therefore appears to represent a fundamental difference in synaptic transmission between the two photoreceptor types. What cellular processes enable rods but not cones to release vesicles at nonribbon sites? One hypothesis suggests that in rods, nonribbon release occurs at random, rather than fixed release sites, in contrast to bipolar cells (Midorikawa et al. 2007; but see Zenisek 2008). In this scenario, membrane-associated components of the SNARE complex (t-SNAREs) might be able to escape ribbon-associated active zones and randomly form ectopic release sites elsewhere on the presynaptic membrane (Chen et al. 2014). If this is the case, why is the SNARE machinery unable to similarly escape the active zone and support nonribbon release in cones? In addition to maintaining a supply of releasable synaptic vesicles, endocytosis is also responsible for clearing used SNARE proteins from presynaptic active zones and rod–cone differences in endocytosis (Van Hook and Thoreson 2012; Cork and Thoreson 2014) could lead to differences in clearance of used SNARE proteins from the ribbon-associated active zone.

### Functional significance

Rod and cone photoreceptors typically have resting potentials of –35 to –40 mV in the dark and are hyperpolarized by a flash of light. This hyperpolarization closes Ca^2+^ channels and reduces the rate of glutamate release. With a transition back to darkness, as simulated by the depolarizing steps employed in this study, Ca^2+^ channel openings trigger a burst of glutamate release that is important in allowing the synapse to encode contrast (Jackman et al. 2009; Oesch and Diamond 2011). This phasic release is followed by a tonic background level of glutamate release that is important in reporting luminance (Jackman et al. 2009; Oesch and Diamond 2011). At many synapses, high levels of fast endogenous Ca^2+^ buffering can help restrict Ca^2+^ to regions near the channels (Nowycky and Pinter 1993; Roberts 1994), thereby promoting synchronous over asynchronous release (Aponte et al. 2008; Eggermann et al. 2012; Kaeser and Regehr 2014). By contrast, in photoreceptors, nanodomain control of release coexists with low levels of endogenous buffering so that both phasic and tonic regimes of exocytosis can operate somewhat independently depending on the timing and amplitude of the stimulus.

This division of release regimes appears to be promoted by the synaptic ribbon, which slows the delivery of vesicles to release sites (Jackman et al. 2009). As a result, although [Ca^2+^] is high around the ribbon and throughout the synaptic terminal, the ribbon itself, by slowing the movement of vesicles toward release sites at the ribbon base, effectively restricts fast exocytosis to nanodomain control so that only vesicles positioned near the membrane and Ca^2+^ domains can be quickly released following Ca^2+^ influx. Later stages of exocytosis are facilitated by low endogenous buffering, which promotes CICR-mediated nonribbon release in rods and CaM-dependent replenishment of ribbons in both rods and cones evoked by spread of Ca^2+^ ions to sites some distance from the channels.

Functionally, sustained release driven by average intraterminal [Ca^2+^] combined with nanodomain-driven fast and efficient release permits each photoreceptor type to report both luminance and temporally precise changes in luminance to postsynaptic neurons, respectively (Jackman et al. 2009; Bartoletti et al. 2011; Oesch and Diamond 2011). At the rod synapse, CICR-mediated nonribbon release makes a substantial contribution to the light responses of postsynaptic retinal neurons (Cadetti et al. 2006; Suryanarayanan and Slaughter 2006; Babai et al. 2010b). The amplification of release rate provided by the actions of CICR likely improves the ability of the rod synapse to report positive contrast since the higher release rates will enhance postsynaptic detection of reduced release associated with light increments (Schein and Ahmad 2005). During sustained depolarization, as occurs in constant darkness, CICR is able to continuously trigger nonribbon release, a process that involves continuous supply of Ca^2+^ via diffusion through the endoplasmic reticulum, which extends from the soma to the terminal (Chen et al. 2014, 2015). Actions of Ca^2+^ to enhance vesicle replenishment and sustained release by ribbons might similarly enhance positive contrast encoding in cone-driven circuits. Additionally, the regulation of IRP replenishment kinetics by Ca^2+^/CaM adds an additional facet to the computations performed at the synapse; by making the size of the IRP dependent on time, the Ca^2+^/CaM-dependent replenishment process also sets the temporal response properties of the synapse and light responses of second-order retinal neurons (Van Hook et al. 2014). In hair cells, Ca^2+^ channel number and voltage dependence appear to vary from one ribbon to the next within single hair cells (Frank et al. 2009). If the same is true for photoreceptors, light offset could be reported differently by each ribbon, possibly contributing to the variability in bipolar cell contrast-response functions (Burkhardt and Fahey 1999; Thoreson and Burkhardt 2003). However, the low Ca^2+^ buffering and resultant spatially unconstrained Ca^2+^ signals would have a normalizing effect across individual ribbons in a given terminal, so that release at neighboring ribbons would be similar during sustained darkness. Finally, expression of Ca^2+^ channels in chick retina is known to vary with circadian time (Ko et al. 2007) and dopamine, a major circadian neuromodulator in the retina modulates Ca^2+^ influx in salamander photoreceptors (Stella and Thoreson 2000; Thoreson et al. 2002). Synaptic ribbon morphology also changes dramatically throughout the day, with ribbons disassembling and becoming detached in the daylight in both mouse and turtle photoreceptors (Abe and Yamamoto 1984; Adly et al. 1999). In fish bipolar cells, diurnal changes in ribbons are associated with changes in Ca^2+^ influx and exocytosis (Hull et al. 2006). Therefore, the combined effects of circadian timing and daily lighting cycles on Ca^2+^ influx and ribbon structure could represent an additional means by which photoreceptor Ca^2+^ signals are regulated to match synaptic signaling requirements. Thus, the exact nature of Ca^2+^ buffering and the means by which it differentially influences each phase of rod and cone synaptic output are likely to play important roles in shaping early stage visual processing at the photoreceptor synapse.

## Conflict of Interest

None declared.

## References

[b1] Abe H, Yamamoto TY (1984). Diurnal changes in synaptic ribbons of rod cells of the turtle. J. Ultrastruct. Res.

[b2] Adler EM, Augustine GJ, Duffy SN, Charlton MP (1991). Alien intracellular calcium chelators attenuate neurotransmitter release at the squid giant synapse. J. Neurosci.

[b3] Adly MA, Spiwoks-Becker I, Vollrath L (1999). Ultrastructural changes of photoreceptor synaptic ribbons in relation to time of day and illumination. Invest. Ophthalmol. Vis. Sci.

[b4] Akopian A, Johnson J, Gabriel R, Brecha N, Witkovsky P (2000). Somatostatin modulates voltage-gated K^+^ and Ca^2+^ currents in rod and cone photoreceptors of the salamander retina. J. Neurosci.

[b5] Aponte Y, Bischofberger J, Jonas P (2008). Efficient Ca^2+^ buffering in fast-spiking basket cells of rat hippocampus. J. Physiol.

[b6] Babai N, Bartoletti TM, Thoreson WB (2010a). Calcium regulates vesicle replenishment at the cone ribbon synapse. J. Neurosci.

[b7] Babai N, Morgans CW, Thoreson WB (2010b). Calcium-induced calcium release contributes to synaptic release from mouse rod photoreceptors. Neuroscience.

[b8] Barnes S, Deschênes MC (1992). Contribution of Ca and Ca-activated Cl channels to regenerative depolarization and membrane bistability of cone photoreceptors. J. Neurophysiol.

[b9] Bartoletti TM, Babai N, Thoreson WB (2010). Vesicle pool size at the salamander cone ribbon synapse. J. Neurophysiol.

[b10] Bartoletti TM, Jackman SL, Babai N, Mercer AJ, Kramer RH, Thoreson WB (2011). Release from the cone ribbon synapse under bright light conditions can be controlled by the opening of only a few Ca^2+^ channels. J. Neurophysiol.

[b11] Baylor DA, Fettiplace R (1977). Kinetics of synaptic transfer from receptors to ganglion cells in turtle retina. J. Physiol.

[b12] Brandt A, Khimich D, Moser T (2005). Few CaV1.3 channels regulate the exocytosis of a synaptic vesicle at the hair cell ribbon synapse. J. Neurosci.

[b13] Bucurenciu I, Bischofberger J, Jonas P (2010). A small number of open Ca^2+^ channels trigger transmitter release at a central GABAergic synapse. Nat. Neurosci.

[b14] Burkhardt DA, Fahey PK (1999). Contrast rectification and distributed encoding By ON-OFF amacrine cells in the retina. J. Neurophysiol.

[b15] Burrone J, Neves G, Gomis A, Cooke A, Lagnado L (2002). Endogenous calcium buffers regulate fast exocytosis in the synaptic terminal of retinal bipolar cells. Neuron.

[b16] Cadetti L, Tranchina D, Thoreson WB (2005). A comparison of release kinetics and glutamate receptor properties in shaping rod-cone differences in EPSC kinetics in the salamander retina. J. Physiol.

[b17] Cadetti L, Bryson EJ, Ciccone CA, Rabl K, Thoreson WB (2006). Calcium-induced calcium release in rod photoreceptor terminals boosts synaptic transmission during maintained depolarization. Eur. J. Neurosci.

[b18] Cadetti L, Bartoletti TM, Thoreson WB (2008). Quantal mEPSCs and residual glutamate: how horizontal cell responses are shaped at the photoreceptor ribbon synapse. Eur. J. Neurosci.

[b19] Chen M, Van Hook MJ, Zenisek D, Thoreson WB (2013). Properties of ribbon and non-ribbon release from rod photoreceptors revealed by visualizing individual synaptic vesicles. J. Neurosci.

[b20] Chen M, Križaj D, Thoreson WB (2014). Intracellular calcium stores drive slow non-ribbon vesicle release from rod photoreceptors. Front. Cell Neurosci.

[b21] Chen M, Van Hook MJ, Thoreson WB (2015). Ca^2+^ diffusion through endoplasmic reticulum supports elevated intraterminal Ca^2+^ levels needed to sustain synaptic release from rods in darkness. J. Neurosci.

[b22] Cho S, Li G-L, von Gersdorff H (2011). Recovery from short-term depression and facilitation is ultrafast and Ca^2+^ dependent at auditory hair cell synapses. J. Neurosci.

[b23] Coggins M, Zenisek D (2009). Evidence that exocytosis is driven by calcium entry through multiple calcium channels in goldfish retinal bipolar cells. J. Neurophysiol.

[b24] Copenhagen DR, Ashmore JF, Schnapf JK (1983). Kinetics of synaptic transmission from photoreceptors to horizontal and bipolar cells in turtle retina. Vision. Res.

[b25] Cork KM, Thoreson WB (2014). Rapid kinetics of endocytosis at rod photoreceptor synapses depends upon endocytic load and calcium. Vis. Neurosci.

[b26] Del Castillo J, Katz B (1954). Quantal components of the end-plate potential. J. Physiol.

[b27] Duncan G, Rabl K, Gemp I, Heidelberger R, Thoreson WB (2010). Quantitative analysis of synaptic release at the photoreceptor synapse. Biophys. J.

[b28] Eggermann E, Bucurenciu I, Goswami SP, Jonas P (2012). Nanodomain coupling between Ca^2+^ channels and sensors of exocytosis at fast mammalian synapses. Nat. Rev. Neurosci.

[b29] Fedchyshyn MJ, Wang L-Y (2005). Developmental transformation of the release modality at the calyx of Held synapse. J. Neurosci.

[b30] Frank T, Khimich D, Neef A, Moser T (2009). Mechanisms contributing to synaptic Ca^2+^ signals and their heterogeneity in hair cells. Proc. Natl. Acad. Sci. U. S. A.

[b31] Heidelberger R, Thoreson WB, Witkovsky P (2005). Synaptic transmission at retinal ribbon synapses. Prog. Retin. Eye Res.

[b32] Helmchen F, Borst JG, Sakmann B (1997). Calcium dynamics associated with a single action potential in a CNS presynaptic terminal. Biophys. J.

[b33] Hidaka S, Christensen BN, Naka K (1986). The synaptic ultrastructure in the outer plexiform layer of the catfish retina: a three-dimensional study with HVEM and conventional EM of Golgi-impregnated bipolar and horizontal cells. J. Comp. Neurol.

[b34] Hull C, Studholme K, Yazulla S, von Gersdorff H (2006). Diurnal changes in exocytosis and the number of synaptic ribbons at active zones of an ON-type bipolar cell terminal. J. Neurophysiol.

[b35] Jackman SL, Choi S-Y, Thoreson WB, Rabl K, Bartoletti TM, Kramer RH (2009). Role of the synaptic ribbon in transmitting the cone light response. Nat. Neurosci.

[b36] Jarsky T, Tian M, Singer JH (2010). Nanodomain control of exocytosis is responsible for the signaling capability of a retinal ribbon synapse. J. Neurosci.

[b37] Kaeser PS, Regehr WG (2014). Molecular mechanisms for synchronous, asynchronous, and spontaneous neurotransmitter release. Annu. Rev. Physiol.

[b38] Ko ML, Liu Y, Dryer SE, Ko GY-P (2007). The expression of L-type voltage-gated calcium channels in retinal photoreceptors is under circadian control. J. Neurochem.

[b39] Korenbrot JI, Rebrik TI (2002). Tuning outer segment Ca^2+^ homeostasis to phototransduction in rods and cones. Adv. Exp. Med. Biol.

[b40] Krizaj D, Bao JX, Schmitz Y, Witkovsky P, Copenhagen DR (1999). Caffeine-sensitive calcium stores regulate synaptic transmission from retinal rod photoreceptors. J. Neurosci.

[b41] Krizaj D, Lai FA, Copenhagen DR (2003). Ryanodine stores and calcium regulation in the inner segments of salamander rods and cones. J. Physiol.

[b42] Krizaj D, Liu X, Copenhagen DR (2004). Expression of calcium transporters in the retina of the tiger salamander (Ambystoma tigrinum). J. Comp. Neurol.

[b43] Lasansky A (1973). Organization of the outer synaptic layer in the retina of the larval tiger salamander. Philos. Trans. R. Soc. Lond. B Biol. Sci.

[b44] Lasansky A (1978). Contacts between receptors and electrophysiologically identified neurones in the retina of the larval tiger salamander. J. Physiol.

[b45] Li W, Chen S, DeVries SH (2010). A fast rod photoreceptor signaling pathway in the mammalian retina. Nat. Neurosci.

[b46] MacLeish PR, Nurse CA (2007). Ion channel compartments in photoreceptors: evidence from salamander rods with intact and ablated terminals. J. Neurophysiol.

[b47] Mehta B, Ke J-B, Zhang L, Baden AD, Markowitz AL, Nayak S (2014). Global Ca^2+^ signaling drives ribbon-independent synaptic transmission at rod bipolar cell synapses. J. Neurosci.

[b48] Mercer AJ, Rabl K, Riccardi GE, Brecha NC, Stella SL, Thoreson WB (2011). Location of release sites and calcium-activated chloride channels relative to calcium channels at the photoreceptor ribbon synapse. J. Neurophysiol.

[b49] Midorikawa M, Tsukamoto Y, Berglund K, Ishii M, Tachibana M (2007). Different roles of ribbon-associated and ribbon-free active zones in retinal bipolar cells. Nat. Neurosci.

[b50] Morgans CW, El Far O, Berntson A, Wässle H, Taylor WR (1998). Calcium extrusion from mammalian photoreceptor terminals. J. Neurosci.

[b51] Neher E, Augustine GJ (1992). Calcium gradients and buffers in bovine chromaffin cells. J. Physiol.

[b52] Nowycky MC, Pinter MJ (1993). Time courses of calcium and calcium-bound buffers following calcium influx in a model cell. Biophys. J.

[b53] Oesch NW, Diamond JS (2011). Ribbon synapses compute temporal contrast and encode luminance in retinal rod bipolar cells. Nat. Neurosci.

[b54] Pang J-J, Gao F, Barrow A, Jacoby RA, Wu SM (2008). How do tonic glutamatergic synapses evade receptor desensitization?. J. Physiol.

[b55] Pangršič T, Gabrielaitis M, Michanski S, Schwaller B, Wolf F, Strenzke N (2015). EF-hand protein Ca^2+^ buffers regulate Ca^2+^ influx and exocytosis in sensory hair cells. Proc. Natl. Acad. Sci. U. S. A.

[b56] Pasino E, Marchiafava PL (1976). Transfer properties of rod and cone cells in the retina of the tiger salamander. Vision. Res.

[b57] Rabl K, Cadetti L, Thoreson WB (2005). Kinetics of exocytosis is faster in cones than in rods. J. Neurosci.

[b58] Rabl K, Cadetti L, Thoreson WB (2006). Paired-pulse depression at photoreceptor synapses. J. Neurosci.

[b59] Rao-Mirotznik R, Harkins AB, Buchsbaum G, Sterling P (1995). Mammalian rod terminal: architecture of a binary synapse. Neuron.

[b60] Roberts WM (1994). Localization of calcium signals by a mobile calcium buffer in frog saccular hair cells. J. Neurosci.

[b61] Sakaba T, Schneggenburger R, Neher E (2002). Estimation of quantal parameters at the calyx of Held synapse. Neurosci. Res.

[b62] Schein S, Ahmad KM (2005). A clockwork hypothesis: synaptic release by rod photoreceptors must be regular. Biophys. J.

[b63] Schnapf JL, Copenhagen DR (1982). Differences in the kinetics of rod and cone synaptic transmission. Nature.

[b64] Singer JH, Diamond JS (2003). Sustained Ca^2+^ entry elicits transient postsynaptic currents at a retinal ribbon synapse. J. Neurosci.

[b65] Snellman J, Mehta B, Babai N, Bartoletti TM, Akmentin W, Francis A (2011). Acute destruction of the synaptic ribbon reveals a role for the ribbon in vesicle priming. Nat. Neurosci.

[b66] Steele EC, Chen X, Iuvone PM, MacLeish PR (2005). Imaging of Ca^2+^ dynamics within the presynaptic terminals of salamander rod photoreceptors. J. Neurophysiol.

[b67] Stella SL, Thoreson WB (2000). Differential modulation of rod and cone calcium currents in tiger salamander retina by D2 dopamine receptors and cAMP. Eur. J. Neurosci.

[b68] Stella SL, Bryson EJ, Thoreson WB (2002). A2 adenosine receptors inhibit calcium influx through L-type calcium channels in rod photoreceptors of the salamander retina. J. Neurophysiol.

[b69] Straiker A, Sullivan JM (2003). Cannabinoid receptor activation differentially modulates ion channels in photoreceptors of the tiger salamander. J. Neurophysiol.

[b70] Suryanarayanan A, Slaughter MM (2006). Synaptic transmission mediated by internal calcium stores in rod photoreceptors. J. Neurosci.

[b71] Szikra T, Krizaj D (2006). The dynamic range and domain-specific signals of intracellular calcium in photoreceptors. Neuroscience.

[b72] Szmajda BA, Devries SH (2011). Glutamate spillover between mammalian cone photoreceptors. J. Neurosci.

[b73] Thanawala MS, Regehr WG (2013). Presynaptic calcium influx controls neurotransmitter release in part by regulating the effective size of the readily releasable pool. J. Neurosci.

[b74] Thoreson WB (2007). Kinetics of synaptic transmission at ribbon synapses of rods and cones. Mol. Neurobiol.

[b75] Thoreson WB, Burkhardt DA (2003). Contrast encoding in retinal bipolar cells: current vs. voltage. Vis. Neurosci.

[b76] Thoreson WB, Nitzan R, Miller RF (2000). Chloride efflux inhibits single calcium channel open probability in vertebrate photoreceptors: chloride imaging and cell-attached patch-clamp recordings. Vis. Neurosci.

[b77] Thoreson WB, Stella SL, Bryson EI, Clements J, Witkovsky P (2002). D2-like dopamine receptors promote interactions between calcium and chloride channels that diminish rod synaptic transfer in the salamander retina. Vis. Neurosci.

[b78] Thoreson WB, Rabl K, Townes-Anderson E, Heidelberger R (2004). A highly Ca^2+-^sensitive pool of vesicles contributes to linearity at the rod photoreceptor ribbon synapse. Neuron.

[b79] Townes-Anderson E, MacLeish PR, Raviola E (1985). Rod cells dissociated from mature salamander retina: ultrastructure and uptake of horseradish peroxidase. J. Cell Biol.

[b80] Van Haesendonck E, Missotten L (1984). Synaptic contacts between bipolar and photoreceptor cells in the retina of *Callionymus lyra* L. J. Comp. Neurol.

[b81] Van Hook MJ, Thoreson WB (2012). Rapid synaptic vesicle endocytosis in cone photoreceptors of salamander retina. J. Neurosci.

[b82] Van Hook MJ, Thoreson WB (2013). Simultaneous whole-cell recordings from photoreceptors and second-order neurons in an amphibian retinal slice preparation. J. Vis. Exp.

[b83] Van Hook MJ, Thoreson WB (2014). Endogenous calcium buffering at photoreceptor synaptic terminals in salamander retina. Synapse.

[b84] Van Hook MJ, Parmelee CM, Chen M, Cork KM, Curto C, Thoreson WB (2014). Calmodulin enhances ribbon replenishment and shapes filtering of synaptic transmission by cone photoreceptors. J. Gen. Physiol.

[b85] Vroman R, Kamermans M (2015). Feedback-induced glutamate spillover enhances negative feedback from horizontal cells to cones. J. Physiol.

[b86] Witkovsky P, Stone S (1983). Rod and cone inputs to bipolar and horizontal cells of the Xenopus retina. Vision. Res.

[b87] Witkovsky P, Schmitz Y, Akopian A, Krizaj D, Tranchina D (1997). Gain of rod to horizontal cell synaptic transfer: relation to glutamate release and a dihydropyridine-sensitive calcium current. J. Neurosci.

[b88] Xu JW, Slaughter MM (2005). Large-conductance calcium-activated potassium channels facilitate transmitter release in salamander rod synapse. J. Neurosci.

[b89] Zampighi GA, Schietroma C, Zampighi LM, Woodruff M, Wright EM, Brecha NC (2011). Conical tomography of a ribbon synapse: structural evidence for vesicle fusion. PLoS ONE.

[b90] Zampini V, Johnson SL, Franz C, Lawrence ND, Münkner S, Engel J (2010). Elementary properties of CaV1.3 Ca^2+^ channels expressed in mouse cochlear inner hair cells. J. Physiol.

[b91] Zenisek D (2008). Vesicle association and exocytosis at ribbon and extraribbon sites in retinal bipolar cell presynaptic terminals. Proc. Natl. Acad. Sci. U. S. A.

[b92] Zenisek D, Steyer JA, Feldman ME, Almers W (2002). A membrane marker leaves synaptic vesicles in milliseconds after exocytosis in retinal bipolar cells. Neuron.

[b93] Zenisek D, Horst NK, Merrifield C, Sterling P, Matthews G (2004). Visualizing synaptic ribbons in the living cell. J. Neurosci.

[b94] Zhang J, Wu SM (2005). Physiological properties of rod photoreceptor electrical coupling in the tiger salamander retina. J. Physiol.

